# Methane Production by Facultative Anaerobic Wood-Rot Fungi via a New Halomethane-Dependent Pathway

**DOI:** 10.1128/spectrum.01700-22

**Published:** 2022-09-14

**Authors:** Xin Huang, Xuan Liu, Yarong Xue, Bingcai Pan, Lei Xiao, Shuijuan Wang, Mark A. Lever, Kai-Uwe Hinrichs, Fumio Inagaki, Changhong Liu

**Affiliations:** a State Key Laboratory of Pharmaceutical Biotechnology, School of Life Sciences, Nanjing University, Nanjing, Jiangsu, China; b State Key Laboratory of Pollution Control and Resource Reuse, School of Environment, Nanjing University, Nanjing, Jiangsu, China; c School of Chemical Engineering and Technology, China University of Mining & Technology, Xuzhou, Jiangsu, China; d Department of Environmental Systems Science, ETH Zürich, Institute of Biogeochemistry and Pollutant Dynamics, Zürich, Switzerland; e MARUM Center for Marine Environmental Sciences, University of Bremen, Bremen, Germany; f Mantle Drilling Promotion Office, Institute for Marine-Earth Exploration and Engineering (MarE3), Japan Agency for Marine-Earth Science and Technology (JAMSTEC), Yokohama, Japan; g Department of Earth Sciences, Graduate School of Science, Tohoku University, Sendai, Japan; State Key Laboratory of Microbial Resources, Institute of Microbiology, Chinese Academy of Sciences

**Keywords:** fungal methane, halomethane-dependent pathway, wood-rot fungi

## Abstract

The greenhouse gas methane (CH_4_) is of pivotal importance for Earth’s climate system and as a human energy source. A significant fraction of this CH_4_ is produced by anaerobic *Archaea*. Here, we describe the first CH_4_ production by facultative anaerobic wood-rot fungi during growth on hydroxylated/carboxylated aromatic compounds, including lignin and lignite. The amount of CH_4_ produced by fungi is positively correlated with the amount of CH_3_Cl produced during the rapid growth period of the fungus. Biochemical, genetic, and stable isotopic tracer analyses reveal the existence of a novel halomethane-dependent fungal CH_4_ production pathway during the degradation of phenol and benzoic acid monomers and polymers and utilization of cyclic sugars. Even though this halomethane-dependent pathway may only play a side role in anaerobic fungal activity, it could represent a globally significant, previously overlooked source of biogenic CH_4_ in natural ecosystems.

**IMPORTANCE** Here, we demonstrate that wood-rot fungi produce methane anaerobically without the involvement of methanogenic archaea via a new, halomethane-dependent pathway. These findings of an anaerobic fungal methane formation pathway open another avenue in methane research and will further assist with current efforts in the identification of the processes involved and their ecological implications.

## INTRODUCTION

Methane (CH_4_) is the most abundant hydrocarbon in Earth’s atmosphere and the second most important greenhouse gas after carbon dioxide (CO_2_), with atmospheric CH_4_ concentrations increasing continually in recent decades (1). Understanding and managing global CH_4_ sources and sinks are thus critical to mitigating anthropogenic climate change. Most CH_4_ in Earth’s atmosphere is of biological origin and believed to be produced anaerobically by groups of *Archaea* that are collectively termed methanogens (2). So far four archaeal methanogenic pathways, all involving the central enzyme methyl coenzyme M reductase (Mcr), have been described. These pathways involve (i) CO_2_ reduction with H_2_ or formate as electron donors (hydrogenotrophic), (ii) fermentation of acetate (aceticlastic), (iii) dismutation of methylated compounds with or without hydrogen (methylotrophic) (2), or (iv) *O*-demethylation of methoxylated aromatic compounds, including lignin derivatives found in coals, coupled to CO_2_ reduction (methoxydotrophic) (3). Methanogenic archaea have been isolated from diverse anoxic environments, such as rice paddy fields, tree trunks, terrestrial and marine sediments, animal gastrointestinal tracts, and coal seams (4). More recently, aerobic pathways of bacterial CH_4_ production by methylthio-alkane reductase through a methionine (Met) biosynthesis pathway (5) and C-P lyase through a phosphonate ester degradation pathway (6, 7) have been described. It was, moreover, shown that cyanobacteria, plants, fungi, animals, including humans, and possibly all living organisms can produce CH_4_ aerobically (8, 9). Yet, the contributions of these aerobic pathways to global CH_4_ pools and emissions are unclear (10).

Lignin, a major, highly cross-linked, phenolic constituent of vascular plants (11), is the second most abundant organic polymer on Earth (12) and accounts for 30% of Earth’s nonfossil organic carbon (13). Biological degradation of lignin plays an important role in the global carbon cycle and production of biofuels (14). Wood-rot fungi, mainly Basidiomycetes, are major biological degraders of lignin and lignite. These fungi produce extracellular oxidases, which degrade the phenolic structure of lignin into phenoxy radicals that can subsequently be degraded by microorganisms (15). Halogen-dependent organic matter breakdown by fungi has been shown previously (16, 17). In addition, wood-rot fungi can produce CH_4_ aerobically (18). The biochemical mechanism of fungal aerobic CH_4_ production is unknown, except that methionine has been proven to be a precursor compound (18). In addition, a few studies have shown that fungi can decompose wood and grass under anaerobic conditions (19). These findings match the cosmopolitan distribution of fungi in anaerobic environments, such as ruminant livestock, anoxic freshwater springs and sediments, wetland soils, and deep subsurface (20). These environments often have large amounts of lignite and high rates of CH_4_ production (21) and atmospheric CH_4_ emissions (22), presenting the possibility that fungi might degrade lignite to energy substrates of methanogenic archaea. The fact that methanogenic archaea are frequently rare, even in sediments where methanogenesis is the dominant respiration pathway (23), moreover, raises the question of whether fungi might be directly involved in the production of this potent greenhouse gas. Due to the tremendous amounts of lignin and lignite coal that are buried under anoxic conditions in nature (24), there is a strong scientific and commercial interest to understand the controls on lignin degradation and the potential to anaerobically convert lignin into biofuels, such as CH_4_.

Here, we demonstrate the anaerobic degradation and conversion of lignite, lignin, phenol, and benzoic acid, as well as their derivatives, to CH_4_ by wood-rot fungi. Through biochemical, genetic, and stable isotopic tracer analyses of Schizophyllum commune 20R-7-F01, we reveal the presence of a novel methanogenic pathway that proceeds via the synthesis of abundant halogenated intermediates in the wood-rot fungi. The detailed mechanism of CH_4_ production by facultative anaerobic wood-rot fungi may represent a significant, previously overlooked source of methane in natural ecosystems.

## RESULTS

### Methane production of *S. commune* 20R-7-F01.

**FIG 1:**
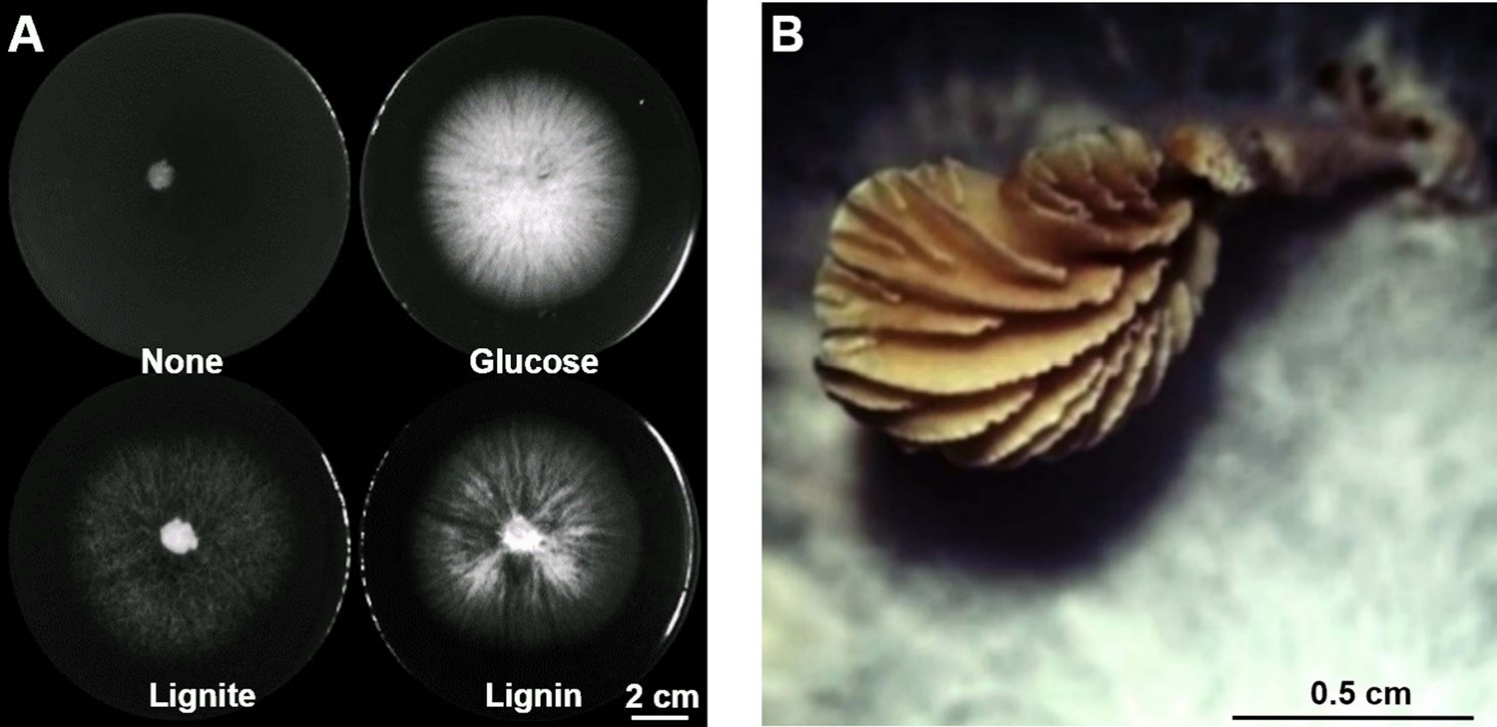
Growth of *S. commune* 20R-7-F01. (A) Anaerobic colonies of strain 20R-7-F01 on agar medium containing glucose, lignite, lignin, and no carbon source at 30°C for 20 days, respectively. (B) Fruiting body of strain 20R-7-F01 on a PDA plate under aerobic conditions.

**FIG 2:**
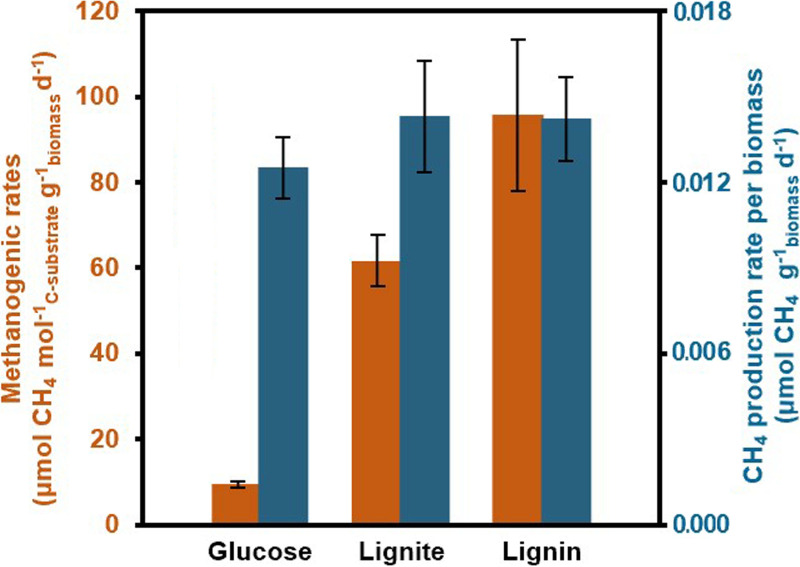
Methanogenic rates of *S. commune* 20R-7-F01 under anaerobic conditions. Shown are the rate of substrate conversion to CH_4_ (orange) (*P* < 0.05) and the rate of CH_4_ production per gram of biomass (blue) (*P* > 0.05) of strain 20R-7-F01 cultured in different substrates for 20 days.

*S. commune* 20R-7-F01 was isolated from ~20-million-year-old deep subseafloor coal-bearing sediment at Site C0020 off the Shimokita Peninsula, Japan, during Integrated Ocean Drilling Program (IODP) Expedition 337 (Fig. 1) (21, 25). To test whether the strain was capable of producing CH_4_, 0.05 g mL^−1^ aerobically grown fungal biomass was inoculated into a 140-mL bottle containing 20 mL of minimal medium (MM) with 2.0 g L^−1^ substrates (glucose, lignin, and lignite) and anaerobically incubated at 30°C for 20 days. The result showed that *S. commune* 20R-7-F01 could produce CH_4_ from lignite, lignin, and glucose under anaerobic conditions with a methanogenic rate that ranged from 9.39 μmol CH_4_ mol^−1^ C-glucose g^−1^ biomass day^−1^ to 95.69 μmol CH_4_ mol^−1^ C-lignin g^−1^ biomass day^−1^ (Fig. 2). Rates were lowest for glucose, which was possibly due to the higher Gibbs energy or higher growth efficiency on glucose than lignin and lignite (Fig. 1A). After 20 days, about 0.008‰, 0.069‰, and 0.044‰ of carbon in glucose, lignin, and lignite are used for CH_4_ release, respectively. The strain has a relatively constant CH_4_ production rate per biomass (0.012 to 0.014 μmol CH_4_ g^−1^ biomass day^−1^) under the given culture condition (Fig. 2). In view of the low CH_4_ production of the fungus, we performed a mass balance experiment using glucose, which showed the lowest conversion rate, to estimate how much of the substrate is transformed into CH_4_. The amount of carbon from glucose consumed by the fungus (893,931.3 nmol) is roughly equal to the carbon transformed into CH_4_ (7.2 nmol), CH_3_Cl (120.0 nmol), CO_2_ (39,115.5 nmol), and biomass (854,666.7 nmol). The conversion rate to CH_4_ was about 0.008‰.

### Isotopic tracer experiment and contamination detection.

**FIG 3:**
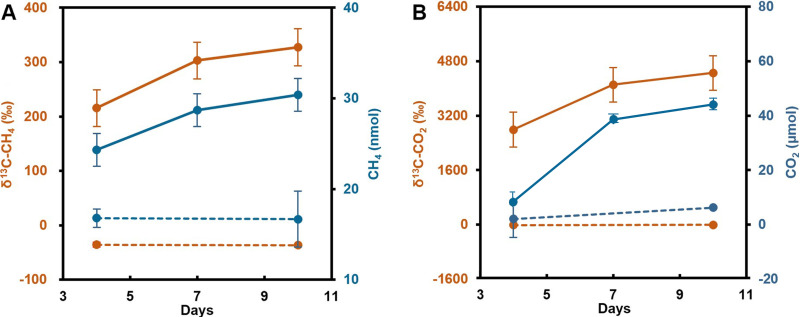
Isotopic evidence for fungus-derived methane formation. Shown are the contents of [δ-^13^C]CH_4_ (A)/[δ-^13^C]CO_2_ (B) (orange solid lines) and total CH_4_ (A)/total CO_2_ (B) (blue solid lines) produced by the strain as well as those that occurred in the strain-free controls (orange and blue dotted lines) in ^13^C-labeled glucose at different culture times.

To confirm that strain 20R-7-F01 can produce CH_4_, we performed a stable isotope tracer experiment with ^13^C-labeled glucose. Gas chromatography and isotope ratio mass spectrometry (GC-IRMS) analysis of the headspace gas showed that the content of CH_4_ was positively correlated with [δ-^13^C]CH_4_ (*R*^2^ = 0.9955, *P* = 0.01) and increased with cultivation time (Fig. 3A). Similarly, CO_2_ content and [δ-^13^C]CO_2_ produced by the strain also increased with culture time (Fig. 3B). Compared with CO_2_, strain 20R-7-F01 produced less CH_4_, and the ratios of total CO_2_ to total CH_4_ generated at days 2, 7, and 10 were 255, 1,392 and 1,488, respectively. No or very little increase in [δ-^13^C]CH_4_ and total CH_4_ (Fig. 3A) as well as [δ-^13^C]CO_2_ and total CO_2_ was observed in the fungus-free control bottles (Fig. 3B), suggesting that the contamination was negligible.

The absence of archaeal and bacterial contaminants was further confirmed by PCR amplification of 16S rRNA and methyl coenzyme M reductase (*mcrA*) genes in the fungal mycelia and culture filtrate (see Fig. S1A in the supplemental material), as well as whole-genome sequencing, which did not yield any recognizable prokaryotic gene sequences (26). In addition, confocal laser scanning microscope (CLSM) observation showed that, except for some general cell internal autofluorescence, no autofluorescence signal (Fig. S1B to K) was found in the filamentous mycelia of strain 20R-7-F01 at an excitation of 405 nm where the emission of methanogenic coenzyme F_420_ would be expected (18, 21). Nucleic acid (Fig. S1B to I) and cell wall (Fig. S1J and K) staining showed internal features of fungal filaments, but no prokaryotic cells were observed. These results confirm that the fungal cultures used in this study were not contaminated by other archaeal and bacterial cells.

### Substrates used for anaerobic fungal methane production.

**FIG 4:**
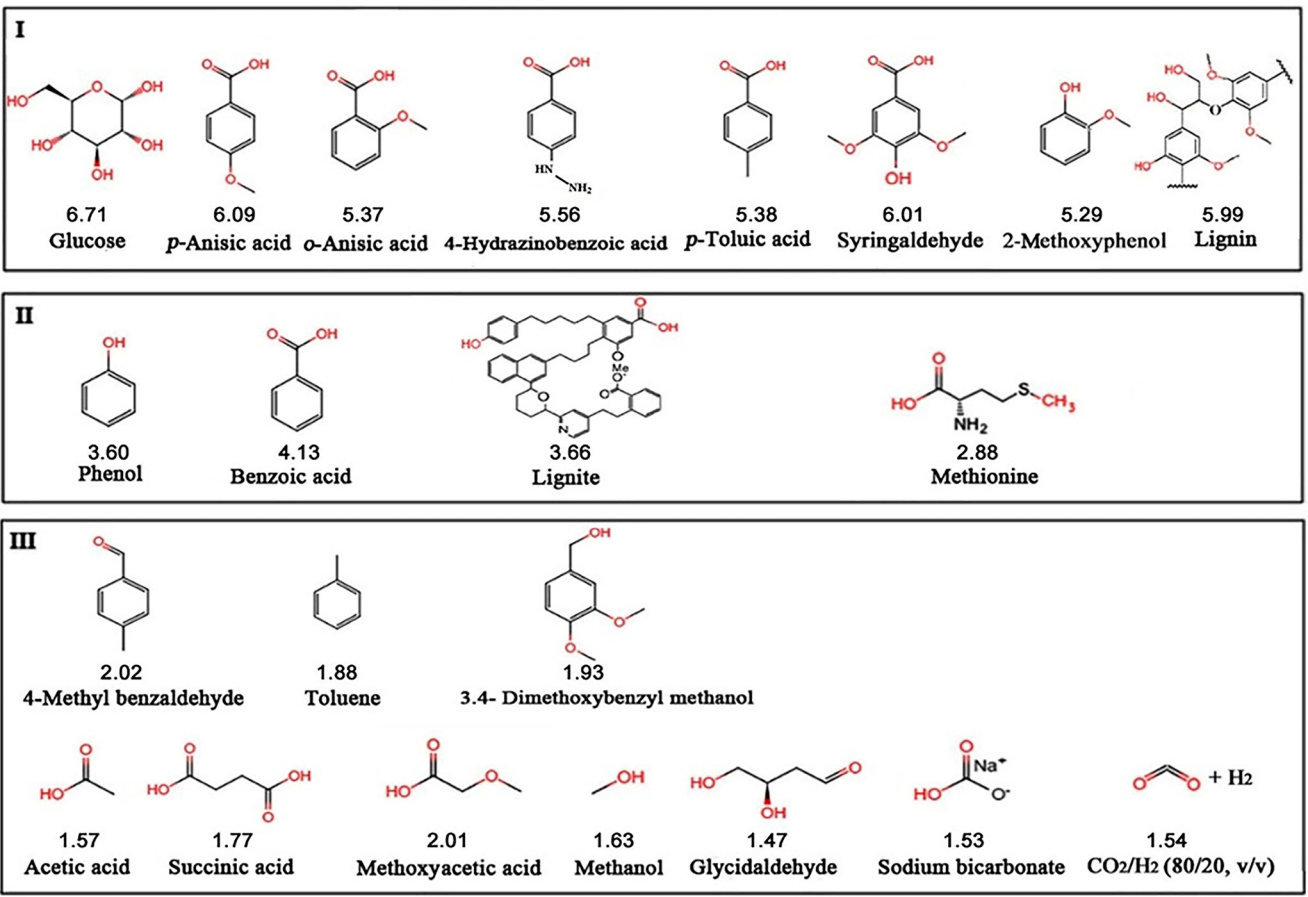
Methane production by *S. commune* 20R-7-F01 in 22 substrates with different structures. The value below each substrate is the average concentration of CH_4_ (nanomoles) produced by the strain using that substrate. The CH_4_ yield produced from group I substrates was significantly higher than that from group II substrates (*P* < 0.05), while the CH_4_ yield in group II substrates was significantly higher than that in group III substrates (*P* < 0.05). The strain cannot produce CH_4_ in group III substrates because it was not significantly different from that of the substrate- and fungus-free controls (1.13 to 1.77 nmol) (*n* = 4). Moreover, the fungal inoculation amount of biomass in each substrate is unified as 0.05 g mL^−1^.

Since *S. commune* 20R-7-F01 was isolated from subseafloor sediment containing lignite (i.e., brown coal), we investigated whether the strain can use lignite and its derivatives to produce CH_4_. The results showed that strain 20R-7-F01 produced CH_4_ from glucose, lignite, lignin, methionine, phenol, 2-methoxyphenol, benzoic acid, 4-hydrazinobenzoic acid, *p*-anisic acid, *p*-toluic acid, *o*-anisic acid, and syringaldehyde (Fig. 4). In contrast, CH_4_ was not produced from linear alkyl acids (i.e., acetic acid, succinic acid, methoxyacetic acid), alcohols (i.e., methanol), or aldehyde (i.e., glycidaldehyde), aromatic compounds lacking phenol and benzoic acid structures, including toluene, 3,4-dimethoxybenzyl methanol and 4-methyl benzaldehyde, and inorganic carbon sources such as CO_2_/H_2_ and NaHCO_3_ (Fig. 4). Based on structural properties and CH_4_ production potential, we grouped the compounds tested into three categories (groups I to III). The largest amount of CH_4_ produced by the strain during the culture period was from group I substrates (5.29 to 6.71 nmol in total), followed by group II substrates (2.88 to 4.13 nmol), both of which were significantly higher than that in group III substrates (1.47 to 2.02 nmol) (*P* < 0.05). Group III substrates did not result in significantly higher CH_4_ yields than fungus-free controls (*P* > 0.05) under the same culture conditions (Fig. 4). Typical substrates of methanogenic archaea (e.g., H_2_/CO_2_, NaHCO_3_, methanol, acetic acid, methoxyacetic acid, and 3,4-dimethoxybenzyl methanol) (2, 3) did not lead to CH_4_ production by the fungus, suggesting that strain 20R-7-F01 produced CH_4_ through a mechanism different from those utilized by archaea.

### The relationship between methyl chloride and methane.

The coupling of phenol and benzoic acid derivative degradation to CH_4_ production (Fig. 4) raises the question of whether methyl chloride (CH_3_Cl) is involved as an intermediate in the CH_4_ production of *S. commune* 20R-7-F01. This is because CH_3_Cl is specifically biosynthesized by wood-rot fungi in the presence of Cl^−^ and acts as a methyl group donor during the methylation of aromatic compounds as part of the lignin degradation process under both aerobic and anaerobic conditions (17). To detect whether CH_4_ production is related to the formation of CH_3_Cl, strain 20R-7-F01 was cultured in six substrates, among which lignin, glucose, and *p*-anisic acid belong to methane-producing substrate group I, lignite belongs to group II, and methanol and benzene belong to non-methane-producing substrate group III. As a result, we found that the strain produces not only CH_4_ but also CH_3_Cl from substrates of both groups I and II (Table S1). The amounts of CH_3_Cl and CH_4_ produced are linearly correlated under the given culture conditions (*R*^2^ = 0.973, *P* = 0.00008). Over a period of 14 days, the average conversions of consumed carbon in glucose, *p*-anisic acid, lignin, and lignite to CH_3_Cl were 0.013%, 0.323%, 0.110%, and 0.061%, respectively, whereas the average conversions of consumed carbon to CH_4_ were 0.008‰, 0.182‰, 0.064‰, and 0.037‰, respectively. The corresponding average ratio of CH_3_Cl to CH_4_ in different substrates is ~17:1. Neither CH_3_Cl nor CH_4_ was produced from substrates of group III. The coincidence of substrate-dependent CH_3_Cl and CH_4_ production strongly suggests that these two products may share the same or closely related metabolic pathway.

**FIG 5:**
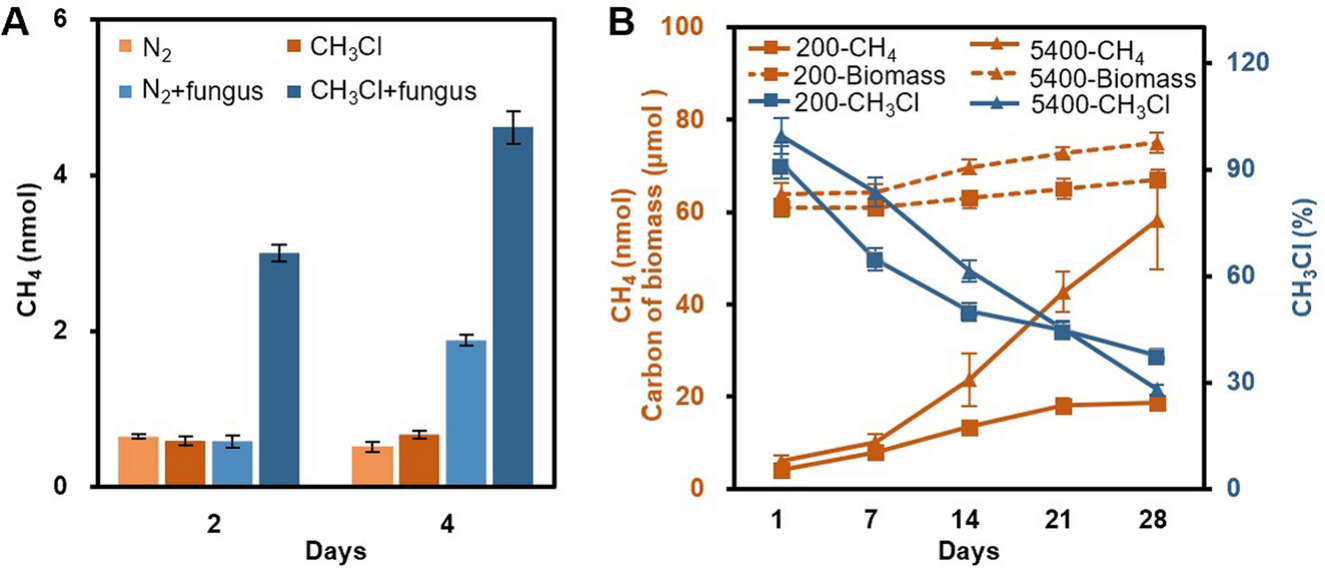
Effect of exogenous methyl chloride on methane production by *S. commune* 20R-7-F01. (A) Effect of exogenous CH_3_Cl on CH_4_ yield 2 and 4 days after inoculation of strain 20R-7-F01 (*n* = 7). The CH_4_ yield of CH_3_Cl treatment (CH_3_Cl+fungus) was significantly higher than those of the other treatments on the 2nd and 4th days after inoculation (*P* < 0.05). (B) Fungal biomass, the amount of residual CH_3_Cl, and the content of CH_4_ produced by strain 20R-7-F01 in medium containing 200 nmol and 5,400 nmol CH_3_Cl within 28 days (*n* = 4). There were no significant differences in CH_4_, CH_3_Cl, and biomass in the controls during the culture period, which is not shown in the figure. The color of the data line corresponds to the color of the vertical axis.

To explore the relationship between fungal CH_3_Cl and CH_4_, 0.05 g mL^−1^ of strain 20R-7-F01 was cultured in a 140-mL anaerobic bottle containing 20 mL *p*-anisic acid (2.0 g L^−1^) medium, supplemented with 1.67 nmol mL^−1^ exogenous CH_3_Cl prepared with nitrogen (200 nmol, less than the maximum CH_3_Cl at 250 nmol in Table S1) at 120 rpm. Pure N_2_ (no CH_3_Cl) was used as a negative control. The results showed that the addition of exogenous CH_3_Cl not only could increase the production of CH_4_ but also could initiate the generation of CH_4_ 2 days earlier than the control (Fig. 5A). This can be explained by the entry of supplemented CH_3_Cl into the cells (16), promoting the formation of CH_4_. Since chloride is an essential reactant for CH_3_Cl biosynthesis (27), we compared the CH_4_ production of strain 20R-7-F01 in the medium with and without halide ions (Cl^−^, Br^−^, and I^−^). Interestingly, the results clearly showed that in comparison to the controls (no fungus, 2.23 nmol), the strain could only produce CH_4_ (6.54 to 7.11 nmol) in the medium containing halides, but not in the medium without halogens (2.89 nmol) (Fig. S2A). These results suggest that halide ions were necessary for the fungus to produce CH_4_. Moreover, we found that the pH of the halide ion medium containing *p*-anisic acid notably decreased from 5.65 to 5.35 (*P* < 0.05), while that of the same halide ion medium containing anisole did not change (5.67 to 5.64; *P* > 0.05) (Fig. S2B), indicating that the halogen- and substrate-dependent production of CH_4_ in the fungus is accompanied by HCl formation.

### Methane derived from methyl chloride.

To determine whether the fungus can directly utilize CH_3_Cl as a carbon source to grow and produce CH_4,_ we conducted two independent experiments. One involved the addition of 125 nmol ^13^C-labeled CH_3_Cl (^13^CH_3_Cl/CH_3_Cl ratio of 1:15, dissolved in liquid methyl *tert*-butyl ether [MBTE]) in 20 mL MM (i.e., 6.25 nmol mL^−1 13^CH_3_Cl) to detect whether ^13^C was converted from ^13^CH_3_Cl to ^13^CH_4_. The other experiment involved the addition of 200 nmol and 5,400 nmol exogenous gaseous CH_3_Cl in a 140-mL anaerobic bottle containing 20 mL MM, respectively, to examine the effect of exogenous CH_3_Cl on CH_4_ production and fungal growth. Both experiments were inoculated at 0.05 g mL^−1^ and cultured at 120 rpm in a shaker. The results clearly showed that ^13^C in CH_4_ produced was indeed derived from ^13^CH_3_Cl (Fig. S2C), and the yield of CH_4_ was related to the contents of exogenous CH_3_Cl (Fig. 5B). At low content, the amount of fungal CH_4_ rapidly increased to 18.11 nmol on the 21st day after inoculation and then remained basically unchanged or slightly increased, while the amount of CH_3_Cl changed just in the opposite direction, decreasing rapidly before 14 days and slowly after that. After 28 days of incubation, 125.05 nmol of exogenous CH_3_Cl was converted to 18.68 nmol of CH_4_ (14.94% of total CH_3_Cl) and 1.80 mg of biomass, and the average production rate of CH_4_ was 0.033 μmol CH_4_ g^−1^ biomass day^−1^. However, at high content, the amount of CH_4_ produced by the strain increased linearly with the extension of culture time, while the amount of exogenous CH_3_Cl consumed by the strain also decreased linearly. A total of 3,888.90 nmol exogenous CH_3_Cl was used by the fungus to produce 58.16 nmol of CH_4_ (1.50% of total CH_3_Cl) and 3.35 mg biomass, and the average production rate of CH_4_ was 0.093 μmol CH_4_ g^−1^ biomass day^−1^. Different contents of exogenous CH_3_Cl have different effects on CH_4_ production and biomass, suggesting that fungi may have different CH_3_Cl metabolism pathways. In addition, no significant decrease of CH_3_Cl and production of CH_4_ was observed in controls without fungus inoculation (data not shown).

### Genes encoding key enzymes for halomethane metabolism.

Given the importance of CH_3_Cl in the formation of fungal CH_4_, we analyzed key genes in the genome of *S. commune* 20R-7-F01 that may be involved in the synthesis and decomposition of CH_3_Cl. It is known that methyl chloride transferase (MCT) is a unique enzyme that catalyzes the synthesis of CH_3_Cl from *S*-adenosyl-l-methionine (SAM) and/or Met in wood-rot fungi (28). SAM/Met is synthesized by methionine synthase (MS) (29), and dehalogenase (DH) is responsible for the degradation of CH_3_Cl (30). Based on the genome analysis, we found that *S. commune* 20R-7-F01 contains a total of two MCT genes (*mct1* and -*2*), five DH genes (*dh1* to -*5*), and one MS gene (*ms*) (Table S2). In addition to *mct*, which is only found in plants (31) and wood-rot fungi (32) including *S. commune* (Fig. S3A), *dh* and *ms* are widely contained in the genomes of both prokaryotic and eukaryotic microorganisms (33, 34) (Fig. S3B and C). Thus, the unique presence of MCT may have given wood-rot fungi the ability to synthesize CH_3_Cl and produce CH_4_. A quantitative real-time PCR (qPCR) with reverse-transcribed RNA analysis also showed that levels of expression of the *mct1*, *ms*, and *dh3*, -*4*, and -*5* genes were apparently upregulated in strain 20R-7-F01 with methanogenic substrates glucose, lignin, phenol, and *p*-anisic acid compared to those in nonmethanogenic substrates methanol and benzene (Fig. S4 and Table S3). In marked contrast, expression of the *mct2* and *dh1* and -*2* genes was not regulated in the strain under the same culture conditions regardless of the substrates (Fig. S4 and Table S3), indicating that these are not involved in the fungal CH_4_ formation process. In addition, compared with the control without addition of CH_3_Cl, the relative expression levels (RQ) of the *dh3* gene in the strain cultured with 5,400 nmol and 200 nmol of exogenous CH_3_Cl were 11.23 and 3.63, respectively (Table S4), suggesting that DH plays an important role in the production of CH_4_ by fungi using CH_3_Cl.

### Methane production by other facultative anaerobic woot-rot fungi.

Given that *S. commune* is a typical wood-rot fungus (35), we wondered whether other wood-rot fungi could also produce CH_4_ via the same methanogenic pathway. Three species of wood-rot fungi with full genome sequences available in the NCBI database were selected, including Agaricus bisporus (36), Hypsizygus marmoreus (37), and Pleurotus ostreatus (38), and anaerobically incubated in *p*-anisic acid and benzene. All three species anaerobically produced both CH_3_Cl and CH_4_ from *p*-anisic acid in comparable amounts to *S. commune*, but not from benzene (Table S5). Not only were *ms*, *mct*, and *dh* genes identified in these fungi, but the expression of these genes was significantly upregulated in the presence of anisic acid compared to benzene (Table S5). These data suggest that facultative anaerobic wood-rot fungi may have a common methanogenic pathway. Moreover, significant fungal laccases activities in the supernatant of medium were detected at 7 days under anaerobic conditions (7.16 to 34.74 mU mL^−1^; *P* < 0.05) (Fig. S5).

## DISCUSSION

To date, it has been demonstrated that organic macromolecule-degrading fungi can produce CH_4_ under aerobic conditions (18) and support anaerobic production of CH_4_ by supplying substrates to methanogenic archaea (39). Yet, the CH_4_ production by facultative anaerobic fungi has never been reported. We for the first time show that *S. commune*, a ubiquitous and well-studied species of wood-rot fungi (35), produces CH_4_ under anaerobic conditions via CH_3_Cl turnover. The dependence of CH_4_ production rates on CH_3_Cl supply (Fig. 4) and the fact that other wood-rot fungi examined can produce CH_4_ with the common key enzymes MCT and DH (see Tables S2 and S5 in the supplemental material) suggest that these fungi may share the same methanogenic pathway. Multiple lines of evidence (e.g., qPCR, CLSM, cultivation, genome sequencing, and mass balance) demonstrate that fungal methanogenic cultures were not contaminated with bacteria, methanogenic archaea, methanogenic endosymbionts, or exogenous carbon.

### The production of fungal methane depends on substrates.

Lignite, also known as brown coal, is a structurally complex intermediate between peat and subbituminous coal and is composed of 3 to 4 effectively stacked aromatic layers and alicyclic layers of hexagonal and pentagonal alicyclic structures (40). The aromatic compounds in lignite and lignin complex often possess hydroxyl, carboxyl, and methoxy substituents directly on benzene rings (41). Among these substituents, methoxy groups are consumed by methylotrophic or methoxydotrophic methanogens to produce CH_4_ (3). Here, we found that *S. commune* 20R-7-F01 is able to produce CH_4_ from glucose, lignite, lignin, methionine, phenol, and benzoic acid as well as their derivatives, but not from well-known substrates of methanogenic archaea, including methoxy groups (2, 3) (Fig. 4). The methane in the cultures of group III and fungus-free controls was most likely derived from the methane adsorbed by the bottles that was reported previously (42). Importantly, all fungal methanogenic substrates (i.e., hydroxyl/carboxyl groups attached to benzene rings [e.g., phenol/benzoic acid derivatives, lignin, and lignite]) and heterocyclic organic compounds (e.g., glucose), are hydrogen donors that are also catabolized by anaerobic microorganisms (43). This suggests that the hydroxyl or carboxyl substituents on heterocyclic compounds and benzene rings may be indispensable in the activation of CH_4_ production. Moreover, besides glucose, Met, and lignin substrates, from which fungi are also known to produce CH_4_ under aerobic conditions (18), fungi growing under anaerobic conditions can additionally use lignite and aromatic compounds such as phenol, benzoic acid, and their derivatives (i.e., 2-methoxyphenol, 4-hydrazinobenzoic acid, *p*-anisic acid, *p*-toluic acid, *o*-anisic acid, and syringaldehyde) to produce CH_4_ (Fig. 4).

### Methyl chloride, a key intermediate for producing methane from specific substrates.

CH_3_Cl is the most abundant volatile halocarbon present in the atmosphere and is mainly derived from wood-rot fungi and plants in marine and terrestrial environments (31, 44). It has been shown that the carbon atom of the sulfur-bound methyl group of Met acts as a precursor of CH_4_ in fungi under aerobic conditions based on isotopic experiments (18). Also, the thiomethyl group of Met is known to be a precursor of CH_3_Cl that is involved as a methyl donor in the biosynthesis of methyl benzoate and methoxyphenol from benzoic acids and phenols by lignin-degrading fungi (45). However, the metabolic link between the production of CH_4_ and CH_3_Cl has not been demonstrated.

In this study, CH_4_ and CH_3_Cl production rates by *S. commune* 20R-7-F01 were positively correlated during the rapid growth period of the fungus and showed the same substrate dependence characteristics (Table S1). The results suggest that CH_4_ is not metabolized by the fungus under anaerobic conditions but CH_3_Cl is an intermediate in the methane formation. Interestingly, the addition of 200 nmol of exogenous CH_3_Cl in the culture bottle containing *p*-anisic acid medium promoted the production of CH_4_ (increased by 2.58 ± 0.16 nmol) (Fig. 5A), and the ^13^C-labeled CH_3_Cl tracer experiment showed that the carbon of CH_4_ was from the carbon of CH_3_Cl (Fig. S2C). These biochemical and isotopic experiments convincingly indicated that the methyl group of CH_3_Cl acts as a precursor of CH_4_ in fungi. In addition, although strain 20R-7-F01 can also produce CH_4_ with Met as the substrate under anaerobic conditions (Fig. 4), it is not clear whether it can directly produce CH_4_ from methionine methyl or whether CH_4_ production from Met goes through CH_3_Cl as an intermediate (28).

Many wood-rot basidiomycetes (e.g., Phanerochaete chrysosporium, Coriolus versicolor, and Phlebia radiate) employ CH_3_Cl as a methyl donor to substrates in intermediary metabolism, without releasing CH_3_Cl during the growth stage (28). On the other hand, Hymenochaetaceae, exemplified by *Phellinus* species (e.g., P. occidentalis, *P. pomaceus*, and *P. ribis*), emit a large amount of CH_3_Cl at the late stage of growth (45). These CH_3_Cl-dependent methylation systems, which stabilize and enhance the activity of fungal oxidative lignin enzymes (28), have been shown to represent a crucial step in the aerobic degradation of lignin by these fungi. It has also been reported that fungi exhibit laccase activity under anaerobic conditions (46) and degrade lignin anaerobically (47). Our study also confirmed that the tested fungi have laccase activity under anaerobic conditions (Fig. S5), but it remains unclear whether this activity is related to degradation of the initial substrate or production of the catabolic intermediate CH_3_Cl. Certain bacteria reportedly transfer methyl groups of CH_3_Cl to tetrahydrofolate and release Cl^−^ by DH (48). However, except for soil bacteria that can degrade dichloromethane and release CH_4_ (49), no microorganism has been identified to degrade halomethanes (CH_3_X, where X represents Cl, Br, and I) to CH_4_. Based on isotope tracer and culture experiments, we found that *S. commune* cannot only produce CH_3_Cl throughout growth (Table S1), but also releases ^13^CH_4_ with ^13^CH_3_Cl as the sole substrate (Fig. S2C). However, the conversion of CH_3_Cl to CH_4_ does not follow a stoichiometric rule, suggesting that the formation of CH_4_ may be a side reaction. Therefore, how fungi use CH_3_Cl to generate energy accompanied with release of CH_4_ remains to be elucidated. In addition, the conversion ratio of exogenous CH_3_Cl to CH_4_ at a high concentration (45 nmol mL^−1^) was only 1.50%, much lower than that at a low CH_3_Cl concentration (14.94% at 1.67 nmol mL^−1^) (Fig. 5B), indicating that a large amount of exogenous CH_3_Cl might be involved in the primary metabolism of fungi as a transition intermediate in the biosynthesis of nonhalogenated products (45).

### Methanogenic pathway of facultative anaerobic fungi.

The key enzymes MCT and DH involved in the production of fungal CH_4_ are ubiquitous in genomes of wood-rot fungi. MCT is a SAM-dependent methyltransferase that is used by fungi and plants and combines the halide ion and methyl of SAM/Met in a nucleophilic substitution reaction to generate CH_3_Cl (28, 50). Two MCT coding genes were identified in *S. commune* 20R-7-F01, and the expression of one gene (*mct1*) had a significant substrate-dependent correlation with the yield of CH_3_Cl and CH_4_ (Fig. S4). *mct1* was also identified in other woot-rot fungi, such as *A. bisporus*, *H. marmoreus*, and *P. ostreatus*, and its expression was significantly upregulated in the presence of the methanogenic substrate *p*-anisic acid (Table S5).

DH can catalyze halogen alkane to form carbon center radicals and halogen anions (30) and is widely produced by many microorganisms (51). We thus propose that DH catalyzes the disproportionation of CH_3_Cl to Cl^−^ and methyl radical (CH_3_·). CH_3_· is a key intermediate in biomethane formation (52). We found that the expression of *dh3* of *S. commune* 20R-7-F01 was significantly upregulated only in the presence of methanogenic substrates (Table S3) and that *dh3* expression levels were significantly correlated with CH_4_ and CH_3_Cl production rates (Fig. S4). Previous studies have suggested that CH_3_· is involved in the methylation of substrates such as benzoic acid and phenols by (*O*-) methyltransferase (53) and combines with H^+^ and e^−^ to form CH_4_ in *S. commune* (54, 55). Moreover, the methylation reaction mediated by DH does not require oxygen, energy, or coenzyme (51), and the synthesis of CH_4_ from CH_3_·, H^+^ and e^−^ is also typically exergonic (56). We therefore hypothesize that the final step of fungal CH_4_ formation proceeds through free radical production by DH, where CH_4_ is a product of CH_3_Cl metabolism. This hypothesis is supported by the results in Fig. 5B, where the ratio of CH_4_ production at high and low CH_3_Cl contents (3.11) matches the ratio of *dh3* RQs at high and low CH_4_ production rates (3.09) (Table S4).

**FIG 6:**
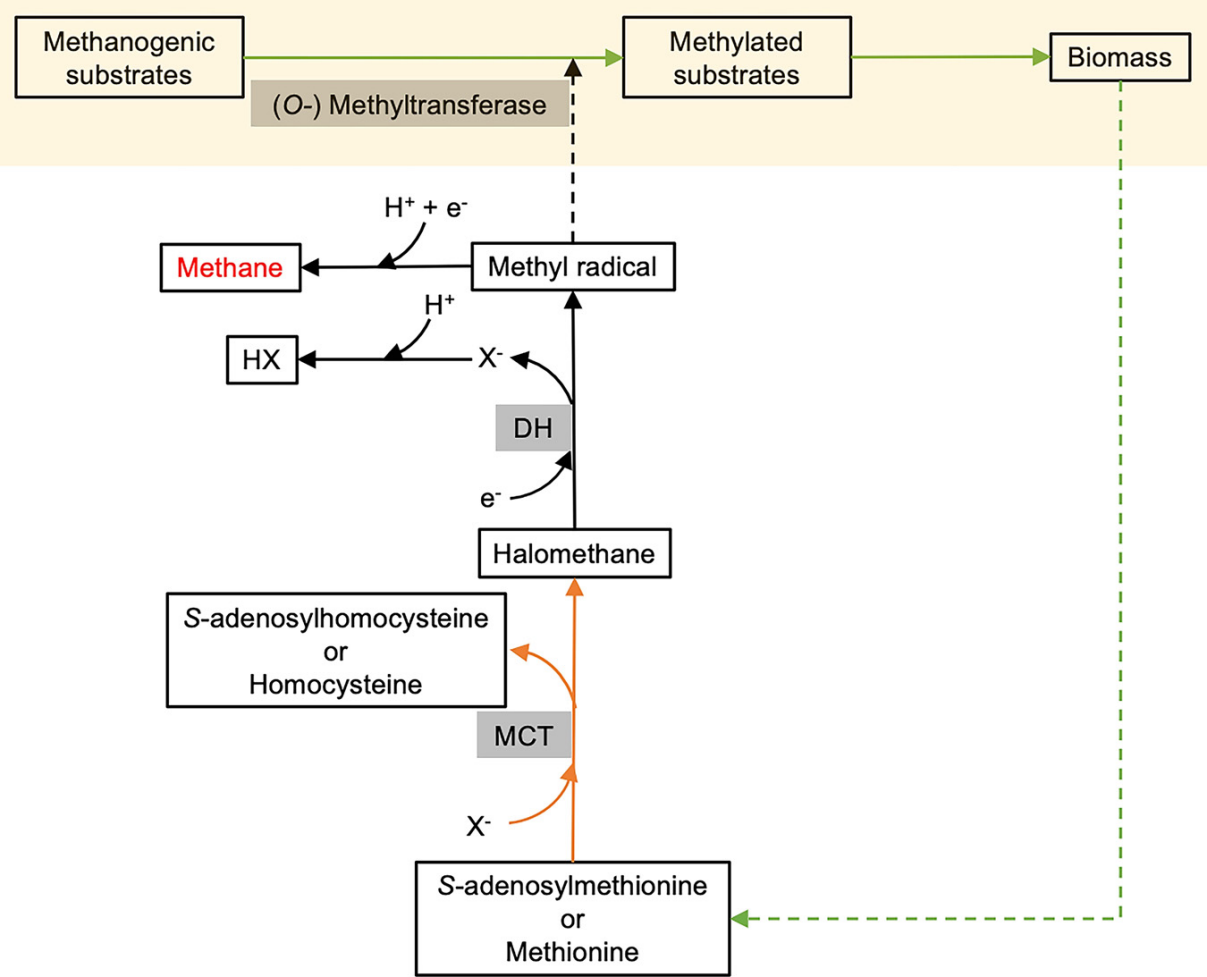
Potential process of halomethane-mediated methane production by facultative anaerobic fungi. Solid lines represent steps I to III. Step I is substrate activation and degradation (green lines), step II is biosynthesis of halomethane (orange lines), and step III is formation of CH_4_ (black lines). X^−^, Cl^−^/I^−^/Br^−^; HX, halogen hydride; MCT, methyl chloride transferase; DH, dehalogenase.

As methanogenesis of *S. commune* is dependent on the synthesis and subsequent breakdown of methyl chloride (Fig. 5, Fig. S2 and S4, and Tables S3 and S4) (28, 30), we propose the involvement of MCT and DH in fungal methanogenesis via a new halomethane-dependent pathway that includes the following steps (Fig. 6).

### (i) Step I: substrate activation and degradation.

It is well known that the key step in the utilization of glucose, benzoic acid, phenol, and their derivatives substrates by wood rot fungi is activation of these substrates (57). Although it is not clear which enzymes and mechanisms are involved in the activation and utilization, laccase has been suggested to play an important role in the anaerobic degradation of (heterocyclic) aromatic compounds such as quinol and ascorbate to produce radicals (46, 57):

benzoic acid/phenol/glucose/their derivatives → (heterocyclic) aryl radicals 

### (ii) Step II: synthesis of halomethane.

Membrane-bound MCT catalyzes the synthesis of CH_3_X from halogen ion and Met/SAM (28, 50):

$${\text{C}}_{5}{\text{H}}_{11}{\text{O}}_{2}{\text{NS/C}}_{15}{\text{H}}_{23}{\text{N}}_{6}{\text{O}}_{5}{\text{S }+\text{ X}}^{-}\to {\text{ CH}}_{3}{\text{X }+\text{ C}}_{4}{\text{H}}_{9}{\text{O}}_{2}{\text{NS/C}}_{14}{\text{H}}_{20}{\text{N}}_{6}{\text{O}}_{5}\text{S}$$

### (iii) Step III: formation of CH_4_.

During dehalogenation by DH, CH_3_X receives e^−^ to produce CH_3_· (30, 49):

$${\text{CH}}_{3}{\text{X}+\text{ e}}^{-}\to {\text{CH}}_{3}\cdot$$

A part of CH_3_· combines with H^+^ and e^−^ (hydrogen abstraction reaction) to form CH_4_ (54, 55):

$${\text{CH}}_{3}{\text{· }+\text{ H}}^{+}{+\text{ e}}^{-}\to {\text{CH}}_{4}$$

### Potential importance of anaerobically produced fungal methane.

Historically, anaerobic archaea have been considered the main biological source of CH_4_ to Earth’s atmosphere. Various bacteria, plants, animals, and wood-rot fungi have also been known to produce CH_4_, albeit aerobically and in much smaller quantities (18). Rather than producing CH_4_ directly, anaerobic fungi have been implicated in the degradation of lignite/lignin to CO_2_, acetate, or alcohol, all of which are substrates of archaeal methane producers (39). We here demonstrate for the first time that anaerobic production of CH_4_ can also be performed directly by wood-rot fungi. These fungi produce CH_4_ during the degradation of lignin/lignite via a novel halomethane-dependent pathway.

Data from this study also indicate that fungal CH_4_ production is not limited to lignin/lignite and their breakdown products, but also takes place with other organic molecules acting as the substrates, including glucose and hydroxylated/carboxylated aromatic compounds (MACs) (Fig. 4 and Table S1). Thus, the spectrum of organic compounds that fuel fungal CH_4_ production could vastly exceed lignin/lignite and their derivatives and exceed the substrate spectrum of aerobic fungal CH_4_ producers (18). Moreover, the genomes of all anaerobic methane-producing fungi contain genes encoding the key enzymes MCT and DH, the expression levels of which increase significantly during fungal CH_3_Cl and CH_4_ production. Given that the functions of MCT and DH are independent of oxygen presence (17, 51), the aerobic production of CH_4_ by wood-rot fungi as reported by Lenhart et al. (18) may adopt the same metabolic pathway as CH_4_ production by facultative anaerobic fungi.

The methanogenic substrates (Fig. 4) of *S. commune* and relatives include building blocks of lignin and carbohydrates (glucose), which are ubiquitous, often dominant components of soil and sedimentary organic matter (14, 58). The widespread distribution of both wood-rot fungi with enzymatic machineries that can be linked to CH_4_ production and known methanogenic substrates of these fungi suggests that fungal CH_4_ production via the halomethane-dependent pathway could be an overlooked, yet globally important phenomenon. Assuming that the global CH_3_Cl emission of fungi is 2 × 10^5^ to 4 × 10^5^ tons year^−1^ (44), and the ratio of CH_3_Cl to CH_4_ produced by fungi is 17:1 to 77:1 (Tables S1 and S5), the global emissions of fungal CH_4_ is estimated to be 2,597 to 23,529 tons year^−1^, which accounts for 0.004 to 0.118% of the global terrestrial CH_4_ emissions (2 × 10^7^ to 6.9 × 10^7^ tons year^−1^) (59). Perhaps, the contribution of fungal CH_4_ to global CH_4_ was overestimated because the production of CH_4_ by fungi is determined under laboratory conditions, but there is no doubt that fungi are at least one of the important contributors of global biogenic methane sources. The quantitative contribution of fungal CH_4_ to biological CH_4_ in anoxic habitats and atmospheric CH_4_ and its role in global climate change need to be further explored.

## MATERIALS AND METHODS

### Fungal species and preservation.

*S. commune* 20R-7-F01 (CGMCC 5.2202) was isolated from deep subseafloor coal-bearing sediments at 1,966.3 meters below seafloor during the IODP Expedition 337 (25). Pleurotus ostreatus bio-52347, Hypsizygus marmoreus bio-089521, and Agaricus bisporus bio-089510 were purchased from the Biobw Culture Collection, Beijing, China. All species were maintained on potato dextrose agar (PDA) medium (potato extract, 200 g L^−1^; glucose, 20 g L^−1^; agar, 20 g L^−1^) at 4°C in our laboratory.

### Culture media.

The medium for methanogenic production (MP medium [pH 6.3 ± 0.1]) consisted of minimal medium (MM) and a certain amount of substrate. MM consisted of basic components (MgCl_2_, 0.5 g L^−1^; KH_2_PO_4_, 1.5 g L^−1^; yeast extract, 0.1 g L^−1^; EDTA, 0.2 g L^−1^) plus trace elements (FeCl_3_, 0.08 g L^−1^; ZnSO_4_, 0.09 g L^−1^; MnSO_4_, 0.03 g L^−1^; CuSO_4_·5H_2_O, 0.005 g L^−1^) and was prepared by adding the filtrate (0.22-μm-pore membrane) of the trace elements into the autoclaved basic component solutions containing a given substrate (121°C for 20 min). The content of the yeast extract was determined according to the specific situation of the experiment; special requirements will be explained separately.

### Inoculation and cultivation.

The growing margin of the fungal colony was taken and homogenized, then transferred to a 500-mL flask containing 200 mL potato dextrose (PD) medium and incubated under aerobic conditions for 5 days at 150 rpm and 30°C. The mycelia in the culture medium were collected by centrifugation at 2,000 × *g* for 20 min and washed with sterile water 3 times as inocula. Except where specifically indicated, 1 g (wet weight) of inoculum was transferred to a 140-mL anaerobic bottle containing 20 mL of the MP medium with a given substrate. To avoid the interference of resazurin as the substrate in the medium to fungal methane and ensure anaerobic cultivation conditions, our study adopted the method of Miller and Wolin (60). Liquid medium prepared by this method remains anoxic, as indicated by resazurin reduction at room temperature for up to 6 months (60). In order to remove oxygen, incubation bottles containing hot, autoclaved media were purged with N_2_ gas (60). Purging continued throughout the subsequent addition of trace element solution, fungal cultures, and sealing, during which an anaerobic test strip (BR0055B; Thermo) fixed inside the headspace part of each bottle (61) turned from pink to white, indicating anaerobic conditions (62) (see Fig. S6A in the supplemental material). To further rule out trace O_2_ requirements of fungal growth, controls containing the O_2_-scavenging reductants iron chloride (FeCl_2_ [aq]; 1 or 2 mM) or ascorbic acid (0.6 mM) were prepared. Addition of chemical reductants had no significant impact on methane production by fungi (Fig. S6B). While methane production also occurred under an oxic headspace (20% O_2_ and 80% N_2_), suggesting that *S. commune* is capable of aerobic methane production, the amount of methane was significantly lower (28.5%; *P* = 0.003, none/aerobic and none/anaerobic) in the presence of O_2_ (Fig. S6B). For further details on the anaerobic cultivation procedure and the experiments to rule out (micro)aerobic fungal methane production, see Text S1 in the supplemental material.

### GC-analysis of methane and methyl chloride.

The concentrations of CH_4_ and CH_3_Cl in the headspace of each bottle were collected by the drainage method, and 1 mL of the gas was measured by GC (HP-Agilent) with a flame ionization detector operated at 300°C with N_2_ as carrier gas (1.3 mL min^−1^) within 10 h after sampling. For CH_4_ measurement, the column HP-PLOT/Q (Agilent, mesh 80/100) was 3.2 m and 1/8 in. in diameter, and detection was performed at 40°C (18). The CH_4_ peak appeared at 2.72 ± 0.1 min, and no CH_3_Cl peak occurred under this detection condition. Moreover, CH_3_Cl was measured using a Porapak-Q column (Supelco) at 130°C (63). The peak of CH_3_Cl appeared at 2.10 ± 0.1 min, and no CH_4_ peak appeared under this detection. The minimal limits of CH_4_ detection and CH_3_Cl detection by GC were 0.05 nmol and 0.14 nmol, respectively (i.e., the upper 95% confidence interval of the negative background).

### Detection of methane production capacity.

To measure the fungal CH_4_ production, *S. commune* 20R-7-F01 was inoculated into three bottles of MM with glucose, lignin, or lignite (2.0 g L^−1^) and incubated at 30°C for 20 days under anaerobic conditions. The same number of uninoculated bottles were used as blank controls for each substrate with three replications (*n* = 3) (Fig. 2). The concentration of CH_4_ in the headspace of each bottle was measured by GC (18), and the remaining glucose, lignin, and lignite in the medium were determined with a d-glucose content assay kit (BoxBio, China), spectrophotometer (64) and dry weight method (65), respectively. The fungal biomass in each bottle was determined after filtration and drying at 65°C for 24 h. The CH_4_ production rate of the fungus was expressed as micromoles of CH_4_ per mole of C-substrate per gram of biomass per day or micromoles of CH_4_ per gram of biomass per day.

To understand what substrates can be metabolized by strain 20R-7-F01 to produce CH_4_, the strain was cultured in 22 substrates (2.0 g L^−1^) of different structures under the anaerobic condition at 30°C for 7 days. Aromatic compounds were prepared by dissolving 40 mg of the chemicals to 0.5 mL ethanol solution and filtered through a 0.22-μm-pore membrane before use. The lignite SH2030 used in this study was from Xinjiang, China, and its vitrinite reflection value (*R*_0_) was 0.49%, containing 68.7% C, 4.1% H, 1.6% S, and 1.2% N. It was homogenized in a mortar and passed through 80- to 100-mesh sieves to remove the large particles. Bottles containing the same amount of MM without substrate and inocula were used as blank controls (CK). The CH_4_ yield of the strain in each substrate was determined by GC. Each substrate was replicated 4 times (*n* = 4) (Fig. 4).

### Mass balance experiment.

The medium used for the mass balance test used MM in which yeast powder was replaced by 0.1 g L^−1^ NH_4_Cl with 2.0 g L^−1^ glucose as the carbon source. After inoculation of *S. commune* 20R-7-F01, three repeated culture bottles were incubated at 30°C for 7 days under anaerobic conditions. The same number of uninoculated bottles were used as blank controls. The concentrations of CH_4_, CH_3_Cl, and CO_2_ in the headspace of each bottle were measured by GC (66), and the remaining glucose in the medium was determined using a d-glucose content assay kit (BoxBio, China). The fungal biomass in each bottle was determined after filtration and drying at 65°C for 24 h. The maximum carbon content of fungal dry weight was calculated as 48% of dry mass by the methods of Bauer et al. (67).

### Stable isotope tracer experiments.

According to the method of Lenhart et al. (18), *S*. *commune* 20R-7-F01 was cultured in nine anaerobic bottles containing MM with 20.0 g L^−1^ glucose (^13^C-labeled glucose/glucose ratio of 1:15) and 2.0 g L^−1^ yeast extract at 30°C. The ^13^C-labeled glucose with 99% isotopic enrichment was purchased from Sigma-Aldrich, Germany. Six bottles without fungus inoculation served as the blank control (CK). The gaseous CH_4_ and CO_2_ in the headspace of the fungus-inoculated bottles were sampled by the drainage method at 4, 7, and 10 days, but that of CK was sampled at 4 and 10 days after inoculation, respectively. All of the gas samples (100 mL per sample) were determined by GC and IRMS (Thermo Fisher) at the same time at the Joint Stable Isotope Laboratory between Shenzhen Huake Precision Analyticals, Inc., and the Tsinghua’ Graduate School at Shenzhen, China. Each treatment has three replications at the sampling time (*n* = 3) (Fig. 3A and B). [δ-^13^C]CH_4_ and [δ-^13^C]CO_2_ were calculated by the following equation (18):

$$[\delta {-}^{13}{\mathrm{C]CH}}_{4}\left([\delta {-}^{13}{\mathrm{C]CO}}_{2}\right)\;\left(‰\right)\;={\text{\{[(}}^{13}{\mathrm{C/}}^{12}{\text{C}}_{\mathrm{sample}}{\text{)/(}}^{13}{\text{C/}}^{12}{\text{C}}_{\mathrm{standard}}\text{)]}\;-\;1\}\times 1,000$$

The same isotopic tracing method was used to detect the C in CH_4_ produced by the fungus from the C in CH_3_Cl, except the substrate was CH_3_Cl. CH_3_Cl purchased from Shanghai Engineering Research Center of Stable Isotope is composed of ^13^CH_3_Cl (with 99% isotopic enrichment) and CH_3_C in a ratio of 1:15 and dissolved in methyl *tert*-butyl ether (MTBE) solution (^13^CH_3_Cl-MTBE). The concentration of CH_3_Cl in MTBE is 1.25 mM. Four treatments were conducted in this experiment, including ^13^CH_3_Cl-MTBE substrate uninoculated with fungus (negative control), fungus without substrate (fungus), MTBE inoculated with fungus (MTBE+fungus), and ^13^CH_3_Cl-MTBE inoculated with fungus (^13^CH_3_Cl+fungus). For the negative-control and ^13^CH_3_Cl+fungus treatments, 0.1 mL ^13^CH_3_Cl-MTBE was added to each bottle of 20 mL MM (the final concentration of CH_3_Cl in the medium was 6.25 nmol mL^−1^), while for the MTBE+fungus treatment, 0.1 mL MTBE was added. Each treatment contains three replications (*n* = 3) (Fig. S2C). The gaseous CH_4_ in the headspace of the anaerobic bottles was measured by GC and IRMS at 7 days after inoculation.

### Tests for microbial contamination.

To eliminate possible contamination of strain 20R-7-F01 by prokaryotes, five specific primer pairs were used to amplify samples collected from ^13^C-labeled glucose tracing experiments after 7 days of incubation. The cetyltrimethylammonium bromide-polyethylene glycol (CTAB-PEG) DNA extraction method was used to extract fungal DNA (68). Genomic DNA used as the positive control was extracted from a pure culture of Bacillus velezensis CC09 and Methanosarcina barkeri DSM800 following the method described in reference 69. Primer pairs targeting bacterial 16S rRNA genes (27F/1429R, ~1,600 bp), archaeal 16S rRNA genes (ARC787F/ARC1059R, ~400 bp), fungal internal transcribed spacer (ITS) (ITS1/ITS4, ~550 bp), and methanogenic *mcrA* genes (MCR-F/MCR-R, ~500 bp; ME1/ME2, ~700 bp) were employed (Fig. S1A). PCR amplification was performed in 50-μL reaction mixtures containing 25 μL 2× *Taq* master mix for PAGE (Vazyme, China), 1 μL forward primer (Tsingeke, China), 1 μL reverse primer, 1 μL template, and 22 μL deionized H_2_O. PCR amplifications were carried out in an SN: 2720500588 thermal cycler (Applied Biosystems) with the procedures described in reference 69. Five microliters of the PCR products was analyzed by 2% agarose gel electrophoresis with staining with Gene Green (Tiangen, China) in 1× TAE (Tris-acetate-EDTA) buffer at 110 V for 35 min (Fig. S1A).

### Confocal laser scanning microscopy.

Fungal cultures derived from glucose isotopic substrate were examined by confocal laser scanning microscopy (CLSM) for general and specific autofluorescence signals and potential extrahyphal bacterial contaminants (Fig. S1B to K). Samples were mounted in 2-mm deep cover well chambers and stained with SYBR green, 4′,6-diamidino-2-phenylindole (DAPI), propidium iodide (PI) (nucleic acids) (Bomei, China), and calcofluor white (cell wall) (MKBio, China). Among them, PI can only stain the nuclei of dead or damaged cells (70). Stains were applied separately. For CLSM analysis, a Zeiss LSM880 (Leica, Heidelberg, Germany) controlled by ZEN black version 2.3 was available. The system was equipped with an upright microscope and a super continuum light source (470 to 670 nm) as well as a 405-nm laser diode. Images were recorded with 60× water immersion lens objective. Data sets were projected by ZEN blue version 2.3.

### Determination of substrate-dependent production of methyl chloride and methane.

To determine the relationship between CH_3_Cl and CH_4_, strain 20R-7-F01 was cultured in MM with four methanogenic (i.e., lignite, lignin, *p*-anisic acid, and glucose) and two nonmethanogenic (i.e., methanol, benzene) substrates (2.0 g L^−1^) at 30°C for 0 (within 2 h), 7, and 14 days, respectively. The same amount of substrate medium without fungal inoculation was used as a blank control (CK). The amount of CH_3_Cl and CH_4_ in headspace of each anaerobic bottle was measured by GC. The net contents were calculated by subtracting the CH_3_Cl and CH_4_ contents in the inoculated bottles from those in the uninoculated bottles. Substrate consumption was calculated by the equation

substrate consumption = substrate content in blank control− substrate content in fungus-inoculated treatments

The residual substrates in the medium were determined by the above method, except for benzene (71) and *p*-anisic acid (72), which were determined by high-performance liquid chromatography (HPLC), and methanol, which was determined with a methanol assay kit (Abcam, China) according to the manufacturer’s instructions. Each treatment has three replications (*n* = 3) (Table S1).

### Effect of exogenous methyl chloride on methane production.

To determine the effect of exogenous CH_3_Cl on CH_4_ production by strain 20R-7-F01, 56 bottles (140 mL) containing 20 mL MM with *p*-anisic acid (2.0 g L^−1^) were flushed with pure N_2_ (purity of 99.999%) for 20 min to remove oxygen and then the following four treatments were performed: (i) the bottle was filled with pure N_2_ but not inoculated with the fungus (i.e., N_2_), (ii) the bottle was filled with 200 nmol CH_3_Cl (38 ppmv CH_3_Cl diluted in pure N_2_ [purchased from Shangyuan Industrial Gas, China]), but not inoculated with the fungus (i.e., CH_3_Cl), (iii) the bottle was filled with pure N_2_ and inoculated with the fungus (i.e., N_2_+fungus), or (iv) the bottle was filled with 200 nmol CH_3_Cl and inoculated with the fungus (i.e., CH_3_Cl+fungus). Each treatment consisted of 14 bottles and was incubated at 30°C. Seven bottles were taken 2 and 4 days after inoculation to determine the content of CH_4_ in the headspace, respectively (*n* = 7) (Fig. 5A).

To investigate the effect of CH_3_Cl on CH_4_ formation, we cultured the strain in a 140-mL anaerobic bottle containing 20 mL MM, which was flushed with pure N_2_ (purity of 99.999%) for 20 min to remove oxygen, and the following six treatments were performed. In treatment 1, the bottle was filled with 200 nmol CH_3_Cl (38 ppmv CH_3_Cl diluted in pure N_2_) but not inoculated with the fungus. In treatment 2, the bottle was filled with 200 nmol CH_3_Cl and inoculated with the fungus. In treatment 3, the bottle was filled with 5,400 nmol CH_3_Cl (1,010 ppmv CH_3_Cl diluted in pure N_2_) but not inoculated with the fungus. In treatment 4, the bottle was filled with 5,400 nmol CH_3_Cl and inoculated with the fungus. In treatment 5, the bottle was filled with pure N_2_ but not inoculated with the fungus. In treatment 6, the bottle was filled with pure N_2_ and inoculated with the fungus. Among them, treatments 1, 3, 5, and 6 were used as the controls. CH_3_Cl was added to each bottle in the same way as with N_2_: i.e., the bottles were flushed with 38 ppmv and 1,010 ppmv CH_3_Cl for 20 min to get the final concentrations of 200 nmol and 5,400 nmol, respectively. CH_3_Cl and CH_4_ contents and biomass in the bottles were measured in treatments 2 and 4 on days 1, 7, 14, 21, and 28, respectively, and in controls on day 1 and 28, respectively. Each treatment contained 4 replicates per sampling time (*n* = 4) (Fig. 5B). The default carbon content was 40% of fungal dry weight. The bottles were incubated in a shaking incubator at 120 rpm to ensure that CH_3_Cl was dissolved in the medium as completely as possible during incubation. The conversion rate of CH_3_Cl to CH_4_ and the fungal CH_4_ formation rate were calculated according to the following equations:

conversion rate of CH3Cl to CH4(%) = (CH4 production/CH3Cl consumption)×100

and

CH4 formation rate (nmol CH4 g−1 biomass day−1) = CH4 production/dry wt of biomass/days

### Effect of halogens on fungal methanogenesis.

Since halogen is a necessary prerequisite for the formation of halomethanes (27), the presence or absence of halogen ions in the medium could indirectly determine whether the fungus can produce CH_4_. To determine whether CH_4_ production is halogen dependent, we compared the differences in the amount of CH_4_ produced by strain 20R-7-F01 in the medium with and without halogen ions. The halogen-free medium was composed of the following: MgSO_4_, 0.5 g L^−1^; KH_2_PO_4_, 1.5 g L^−1^; EDTA, 0.2 g L^−1^; syringaldehyde, 2.0 g L^−1^; NH_4_Fe(SO_4_)_2_ 12H_2_O, 0.24 g L^−1^; ZnSO_4_, 0.09 g L^−1^; MnSO_4_, 0.03 g L^−1^; CuSO_4_·5H_2_O, 0.005 g L^−1^. The halogenated medium was prepared by adding 0.1 g of KCl/KBr/KI to the halogen-free medium. Strain 20R-7-F01 was cultured in the halogen-free and halogenated media (i.e., KCl, KBr, and KI) at 30°C for 7 days. Each treatment had three bottles. Three bottles containing halogen-free medium and uninoculated the fungus were used as the blank control (i.e., negative control), which was incubated under the same culture conditions. The content of CH_4_ produced by the strain in the headspace of each bottle was measured by GC (*n* = 3) (Fig. S2A).

### Effects of methane-producing fungus on pH change of culture medium.

To determine whether CH_4_ production leads to pH decline, strain 20R-7-F01 was cultured in MM containing the methane-producing substrate of *p*-anisic acid and non-methane-producing substrate of anisole (2.0 g L^−1^) at 30°C for 2, 7, and 14 days, respectively. The content of CH_4_ in the headspace of the anaerobic bottles (i.e., anisic acid-CH_4_, anisole-CH_4_) and the pH of the medium (i.e., anisic acid pH, anisole pH) were determined with a benchtop pH meter (REX, China). Each treatment had three bottles (*n* = 3) (Fig. S2B).

### Identification and quantification of genes related to fungal methanogenesis.

Phylogenetic analysis of MCT, MS, and DH genes extracted from NCBI database was carried out in this study using distance and parsimony methods (1,000 bootstraps) implemented in PAUP version 4.0b10 (Fig. S3) (73). Strain 20R-7-F01 was cultured anaerobically at 30°C for 7 days in MM containing 0.2 g L^−1^ benzene, methanol, phenol, *p*-anisic acid, lignin, and glucose. The same amount of substrate medium without fungal inoculation was used as a blank control (CK). Total RNA was extracted from each of the above samples using TRIzol reagent (Tiangen, China) and treated with DNase I (TaKaRa, Japan) according to the manufacturer’s protocol (74). The SYBR Premix ExTaq II kit (TaKaRa) and gene-specific primer pairs (Table S2) were used for qPCR. Moreover, *act1* and *tef1* were used as reference genes for *S. commune* 20R-7-F01 with 4 repeats per substrate (*n* = 4) (Table S3 and Fig. S4). qPCR amplification was performed as follows: 95°C for 30 s, followed by 45 cycles of 95°C for 5 s, and 55°C for 15 s. Relative expression levels of genes (RQ) in methane-producing substrates (e.g., phenol, *p*-anisic acid, lignin, and glucose) and non-methane-producing substrates (e.g., benzene/methanol) were evaluated by the threshold cycle (2^−ΔΔ^*^CT^*) method (75). When 2^−Δ^*^CT^* values have a significant difference (*P* < 0.05), an RQ of >2 or <0.5 indicates that the expression of the given gene is significantly upregulated or downregulated between two samples (64). The results of RQ were analyzed by false-discovery rate (FDR) to determine its reliability (76). The FDRs of our study were all ≤7.69% (i.e., positive predictive value greater than 90% confidence interval). FDR is calculated as follows:

FDR = FP/(FP + TP) = 1 − PPV

where FP is false positive with an RQ of >2 and a *P* value of ≥0.05, TP is true positive with an RQ of >2 and *P* value of <0.05, and PPV is the positive predictive value and often referred to as precision.

### Ability of other facultative anaerobic wood-rot fungi for methyl chloride and methane production.

To test whether other wood-rot fungi have the same methanogenic capacity and pathway as *S. commune*, three fungi, including *P. ostreatus*, *H. marmoreus*, and *A. bisporus* were selected and cultured in methanogenic substrate *p*-anisic acid and nonmethanogenic substrate benzene (0.2 g L^−1^) at 25°C (their optimal temperature) for 7 days (77). The CH_3_Cl and CH_4_ produced by these fungi in the headspace of the anaerobic bottles were measured by GC. Genes encoding MCT, MS, and DH were identified from these fungi according to their genome sequences (*P. ostreatus*, QLNW00000000.2; *H. marmoreus*, JABWDO000000000.1; and *A. bisporus*, AEOK00000000.1) in the NCBI database (Table S2), and their levels of expression in methanogenic and nonmethanogenic substrates were quantified by qPCR as described above. Moreover, *act1* was used as reference genes for these fungi with 6 repeats per substrate for each fungus (*n* = 6) (Table S5).

### Determination of fungal anaerobic laccase activity.

To determine whether fungi can produce laccase under anaerobic conditions, *S. commune*, *P. ostreatus*, *H. marmoreus*, and *A. bisporus* were anaerobically cultured in 140-mL anaerobic bottles containing 20 mL MM and 2.0 g L^−1^ *p*-anisic acid at 30°C at 120 rpm for 7 days. MM containing *p*-anisic acid without fungal inoculation was used as a control (CK). Each treatment had 3 replicates (*n* = 3) (Fig. S5A). In the anaerobic chamber, the fungal laccase anaerobic activity was measured by using ABTS (laccase kit) (Solarbio, China) as the substrate, which is typically and widely used for the determination of laccase activity (78). Basically, the operation of laccase activity detection was performed as follows in an anaerobic chamber: (i) the *p*-anisic acid medium was centrifuged with a palm centrifuge for 10 min to obtain supernatant, (ii) 150 μL of the supernatant was mixed with 850 μL working solution containing ABTS and incubated at 45°C for 3 min, (iii) OD values of the mixture before and after 3 min were detected separately at 420 nm, and (iv) laccase anaerobic activity was calculated according to the difference in OD values. One unit of enzymatic activity (U) was defined as the amount of enzyme catalyzing the oxidation of 1 μmol of ABTS in 1 min. Moreover, in order to further confirm that fungi can secrete laccase and have activity under anaerobic conditions, we inoculated *S. commune* on the guaiacol-containing medium for observation. Laccase can oxidize colorless guaiacol to brown. The controls were the guaiacol-containing mediums not inoculated with fungus. Each treatment contained three replications (*n* = 3) (Fig. S5B). The specific operations were as follows: (i) mycelia growing on the edge of colony on PDA medium under aerobic conditions were placed in the center of guaiacol-containing medium, (ii) the guaiacol-containing medium was inoculated with fungal mycelia, and the controls were placed in an anaerobic chamber for culture at 30°C, and (iii) the color change of guaiacol-containing medium was observed every day. The guaiacol medium consisted of the following: 2-methoxyphenol (also known as guaiacol), 0.04%; peptone, 2.6 g L^−1^; MgSO_4_ 7H_2_O, 0.5 g L^−1^; KH_2_PO_4_, 1.0 g L^−1^; Na_2_HPO_4_, 0.2 g L^−1^; agar, 20.0 g L^−1^.

### Data availability.

Genes encoding MCT, DH, and MS in *S. commune* 20R-7-F01 genome (accession no. VCHW00000000) were identified by gene annotation and homology comparative analysis (79). The eight genes related to methane formation by facultative anaerobic fungi are as follows: MCT, unitig_415.g125 and unitig_415.g126; MS, unitig_19.g239; and DH, unitig_21.g242, unitig_19.g451, unitig_20.g121, unitig_20.g125, and unitig_30.g52. The sequences have been uploaded to GenBank under accession no. OM864274, OM864275, OM864268, OM864272, OM864269, OM864270, OM864271, and OM864273, respectively (Table S2). All data are available in the main text or supplemental material (80–83).
