# Supplementary material for: Methane Production by Facultative Anaerobic Wood-Rot Fungi via a New Halomethane-Dependent Pathway
Source: Microbiol Spectr. 2022 Sep 14;10(5):e01700-22. doi: 10.1128/spectrum.01700-22 (PMC9604129; doi:10.1128/spectrum.01700-22)
Supplement: Supplemental file 1 — Supplemental material. Download spectrum.01700-22-s0001.pdf, PDF file, 0.8 MB [file spectrum.01700-22-s0001.pdf]

SUPPLEMENT FIGURE

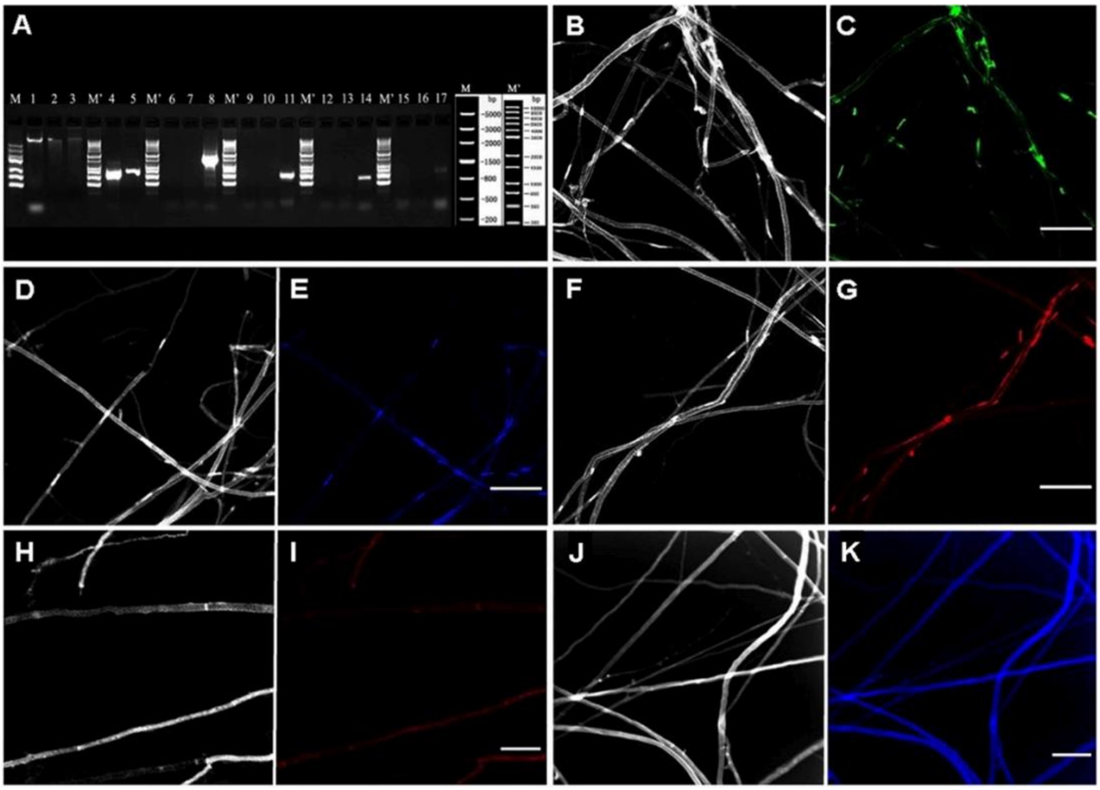

Fig. S1. Exclusion of contamination of *S. commune* 20R-7-F01 by other microorganisms. **(A)** PCR products of *S. commune* 20R-7-F01 cultivation system. M, DNA Marker III. 1, genome DNA of strain 20R-7-F01 (fungus). 2, genome DNA of *Methanosarcina barkeri* DSM800 (archaea). 3, genome DNA of *Bacillus velezensis* CC09 (bacteria). M', 1 kb plus Marker. 4, ITS fragment of fungus. 5, ITS fragment of culture filtrate. 6, 16S rRNA (bacteria) fragment of fungus. 7, 16S rRNA (bacteria) fragment of culture filtrate. 8, 16S rRNA (bacteria) fragment of bacteria. 9, 16S rRNA (archaea) fragment of fungus. 10, 16S rRNA (archaea) fragment of culture filtrate. 11, 16S rRNA (archaea) fragment of archaea. 12, *mcrA* fragment (ME primers) of fungus. 13, *mcrA* fragment (ME primers) of culture filtrate. 14, *mcrA* fragment (ME primers) of archaea. 15, *mcrA* fragment (MCR primers) of fungus. 16, *mcrA* fragment (MCR primers) of culture filtrate. 17, *mcrA* fragment (MCR primers) of archaea. **(B~K)** Confocal laser-scanning microscopy of *S. commune* 20R-7-F01. Maximum intensity projection of fungal filaments from *S. commune*. Staining with different nucleic acid-specific fluorochromes is shown for sybrGreen **(B)**, 4′,6-diamidino-2-phenylindole (DAPI) **(D)**, Propidium iodide (PI) **(F, H)** and cell wall-specific fluorochromes Calcofluor White **(J)**. To present the fluorescence emission signals with highest contrast, they are presented as measured by the photomultiplier in 8-bit grey scale. For clarity the reflection

signal recorded for the samples in **(B)** was removed. **(C, E, J, I, K)** Maximum intensity projection of *S. commune* cultures including the reflection signal **(C)**. Staining is shown for different nucleic acid-specific fluorochromes including sybrGreen **(C)**, DAPI **(E)**, PI **(G, I)** and Calcofluor White **(K)**. Color allocation: **(C)** sybrGreen=green, reflection=grey; **(E)** DAPI=blue; **(G, I)** PI=red. **(F, G)** are the inactivated fungal filaments that heat by the flame. **(H, I)** are the living fungal filaments. PI cannot stain the nucleus of the intact cells. Please notice the heterogenic distribution of the nucleic acid signal, which is characteristic for many fungal strains. In addition, it is obvious that no bacterial signal is visible, neither as single cell, microcolony or associated with fungal filaments. Scale bars; 20 µm.

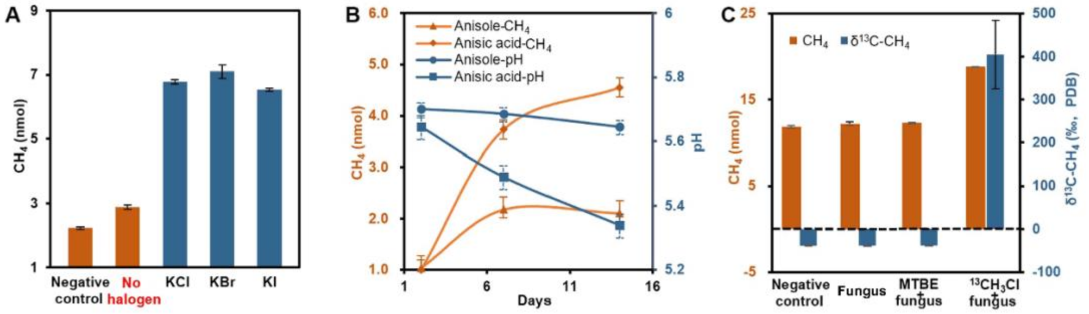

Fig. S2. Relationship between methyl chloride and methane production by *S. commune* 20R-7-F01. (A) Role of halogens, as an essential factor for methyl chloride methyl chloride biosynthesis, in the anaerobic methanogenesis of strain 20R-7-F01. The CH_4 content in the medium with halogens and fungus was significantly higher than that of negative control (no fungus) and no halogen (with fungus) ($p<0.05$) (n=3). **(B)** The amount of CH_4 produced by the strain in the headspace of the anaerobic bottles and the pH value of the medium at different days after inoculation. Statistical analysis showed that the CH_4 yield of the strain was significantly increased ($p<0.05$) and the pH value was significantly decreased ($p<0.05$) when cultured in *p*-anisic acid. However, there was no significant change in CH_4 content and pH value when cuAltured in anisole ($p>0.05$) (n=3). **(C)** The contents of $\delta^{13}C$-CH_4 and total CH_4 produced by the strain in ^{13}C-labeled CH_3Cl substrate 7 days after inoculation. The amount of CH_4 in ^{13}C-labeled CH_3Cl treatment ($^{13}CH_3Cl$+fungus) was significantly higher than those of the control (Negative control; Fungus; MTBE+fungus) (n=3).

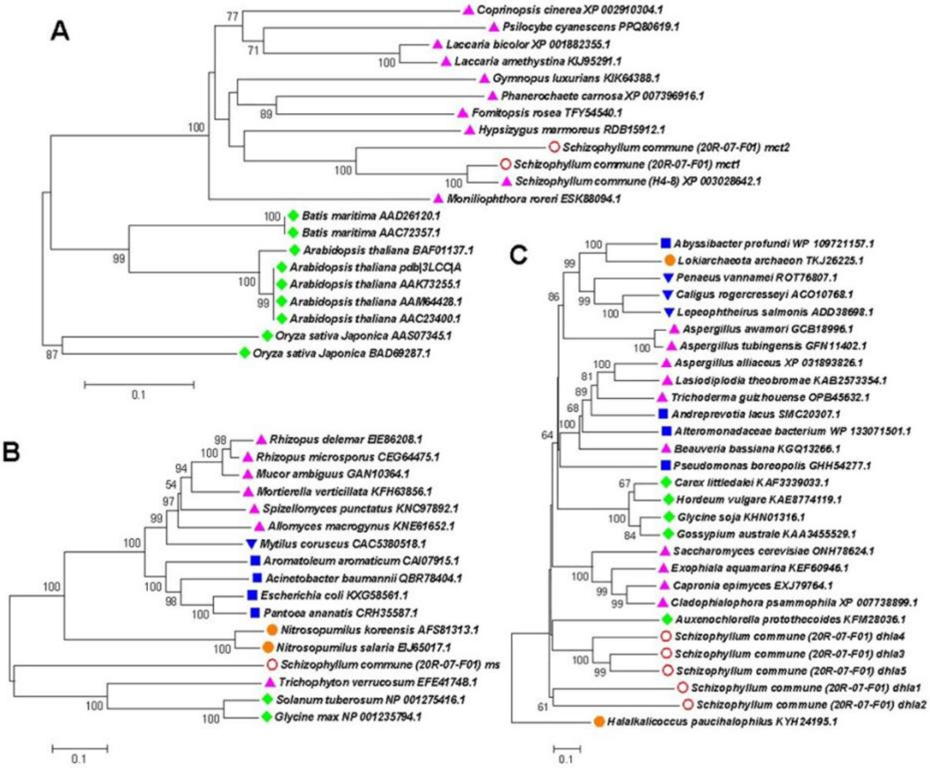

Fig. S3. Phylogeny of methanogenesis-related genes in *S. commune* 20R-7-F01. NJ tree constructed based on protein sequences of *mct* **(A)**, *ms* **(B)**, and *dh* **(C)** of all organisms deposited in NCBI database. The absence of a number indicates the confidence coefficient <50. ▲, ▼, ♦, ■, ●, and ○ are symbols for the discovery of these protein sequences in fungi, animals, plants, bacteria, archaea and *S. commune* 20R-7-F01, respectively. MCT encoding genes are found only in plant and wood rot fungi (**A**), while MS (**B**) and DH (**C**) encoding genes are widespread in the genomes of prokaryotes and eukaryotes.

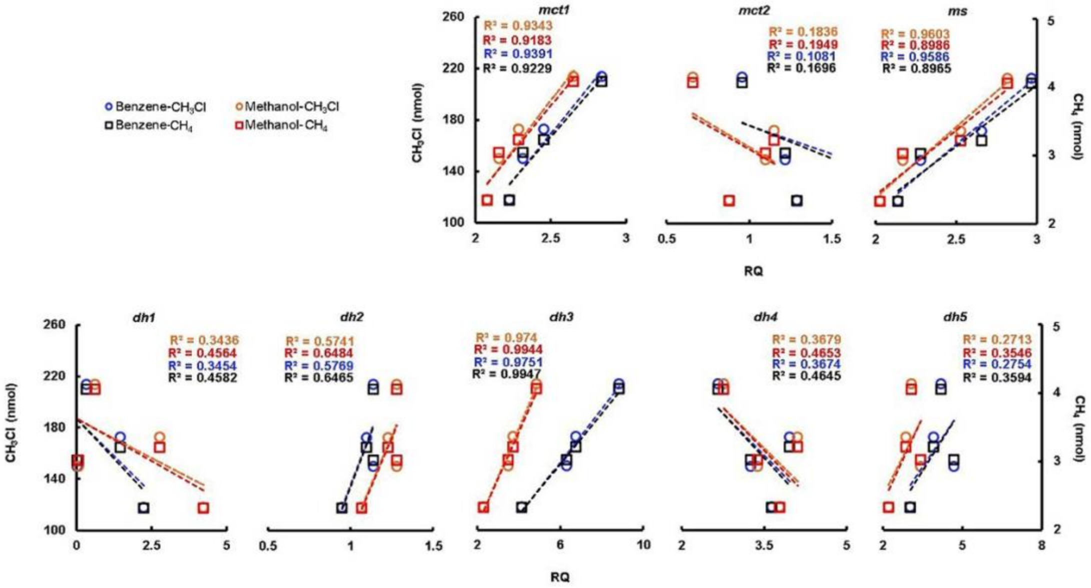

Fig. S4. Correlation analysis of RQ of *mct1*, *mct2*, *ms*, *dh1-5* genes, and the amount of CH_3Cl and CH_4. CH_3Cl, circle symbol; CH_4, square symbol. The RQ value was calculated by the $2^{-\Delta\Delta CT}$ method (75), representing the expression of these genes in methanogenic substrates (glucose, lignin, phenol, and *p*-anisic acid) relative to that in non-methanogenic substrates methanol and benzene, respectively (n=4). Among the different methanogenic substrates, the relative expression of *mct1*, *ms*, and *dh3* showed a significant substrate-dependent correlation with the corresponding yield of CH_3Cl and CH_4, respectively.

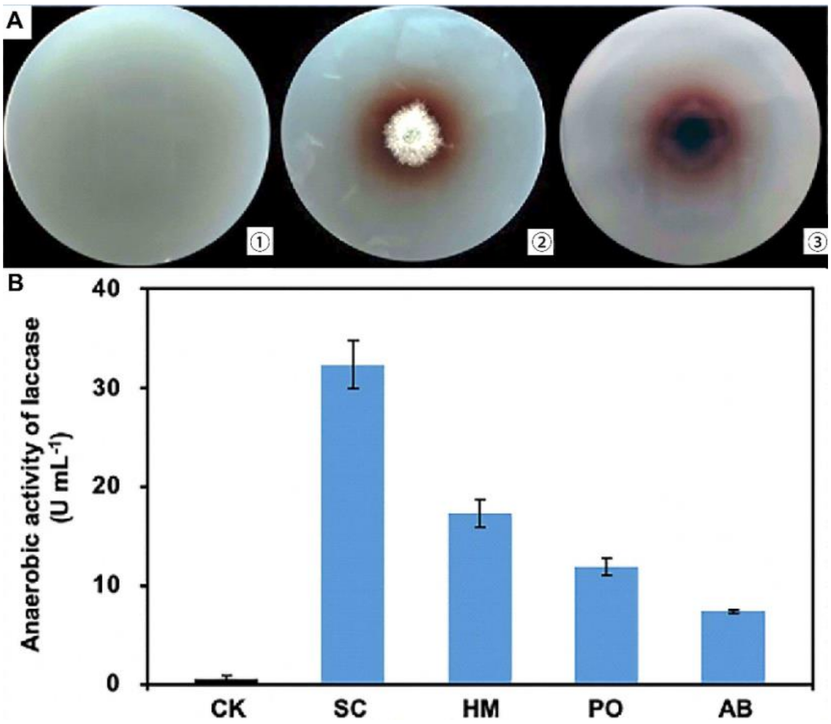

Fig. S5. Detection of laccase activity in fungi under anaerobic conditions. (A) Laccase activity of strain 20R-7-F01 on guaiacol-containing medium. From left to right are fungal-free control, front and back of the petri dish at 3 days of anaerobic incubation. (B) Laccase activity of woot rot fungi in MM meium containing *p*-anisic acid at 7 days of anaerobic incubation. CK: medium without fungal inoculation (controls); SC: *S. commune*, PO: *P. ostreatu*, HM: *H. marmoreus*, AB: *A. bisporu*.

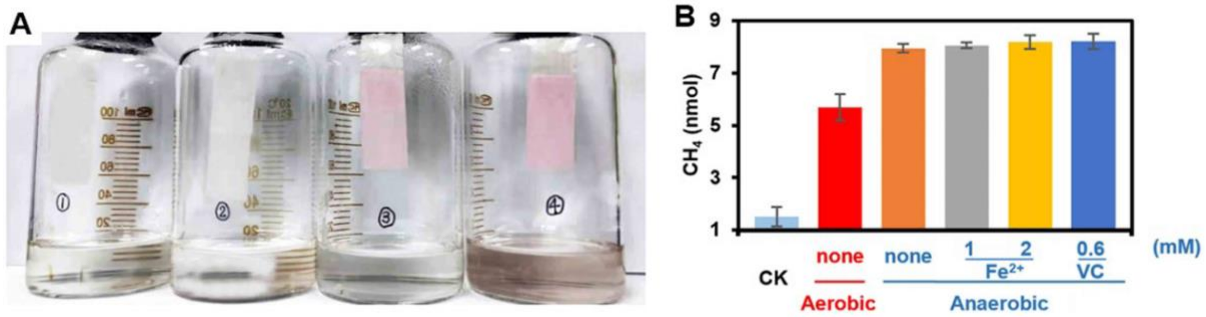

Fig. S6. Confirmation of anaerobic culture conditions. (A) Comparison between adding resazurin into MP medium and using resazurin containing test strip to indicate anaerobic conditions. ① medium + 0.1% resazurin + N_2 purging for 10 minutes (test strip: white; medium: colorless); ② medium + N_2 purging for 10 minutes (test strip: white; medium: colorless); ③ medium only (test strip: pink; medium: colorless); ④ medium + 0.1% resazurin (test strip: pink, medium: pink). The results clearly show that the test strip has the same sensitivity to oxygen as the addition of resazurin in the culture medium, and can be used to indicate whether the fungal culture environment is anaerobic. **(B)** Effect of reducing agent and oxygen on CH_4 production by *S. commune* 20R-7-F01. Fungi were grown in MP medium with 2.0 g L^{-1} glucose for 7 days under aerobic (20% O_2, 80% N_2) and anaerobic conditions (no reductant ("none"), 1 and 2 mM $FeCl_2$ as reductant, 0. 6mM ascorbic acid as reductant). CK represents medium without fungi. Aerobic: bottles flushed with a 20% O_2/80% N_2 gas mixture for 20 min. Anaerobic: bottles flushed with pure N_2 (99.999%) for 20 min. VC represents ascorbic acid. Bars indicate averages and standard deviations of three replicate incubations.

SUPPLEMENT TABLE

Table S1. The yields of CH_4, CH_3Cl, biomass and the remaining substrate in the anaerobic bottles inoculated with strain 20R-7-F01 for 0, 7 and 14 days respectively.

Substrate	Days	CH_4 (nmol)			CH_3Cl (nmol)			Residual carbon (mmol)			Biomass (mg)		
		Flask 1	Flask 2	Flask 3	Flask 1	Flask 2	Flask 3	Flask 1	Flask 2	Flask 3	Flask 1	Flask 2	Flask 3
***p*-Anisic acid**	**0**	0.00	0.00	0.00	0.00	0.00	0.00	2.10	2.10	2.10	13.70	11.70	11.00
	**7**	4.50	3.59	4.71	72.32	59.09	77.09	2.06	2.07	2.07	28.00	33.00	29.00
	**14**	9.59	8.73	6.70	132.96	147.21	164.46	2.06	2.06	2.06	32.00	33.00	38.00
**Glucose**	**0**	0.00	0.00	0.00	0.00	0.00	0.00	1.33	1.33	1.33	11.00	13.70	11.70
	**7**	7.45	8.41	8.14	146.57	108.05	132.43	0.34	0.42	0.45	37.50	34.50	39.30
	**14**	10.50	8.63	11.25	178.50	150.91	172.13	0.00	0.00	0.00	43.00	39.00	41.00
**Lignin**	**0**	0.00	0.00	0.00	0.00	0.00	0.00	2.38	2.38	2.38	11.00	13.70	11.70
	**7**	4.98	4.77	5.84	117.00	101.46	125.52	2.24	2.24	2.25	28.50	29.80	29.20
	**14**	9.21	9.05	9.43	160.07	166.29	144.96	2.14	2.15	2.15	35.30	35.60	34.30
**Lignite**	**0**	0.00	0.00	0.00	0.00	0.00	0.00	2.29	2.29	2.29	11.00	13.70	11.70
	**7**	4.61	3.64	4.18	103.66	63.70	110.84	2.12	2.11	2.09	27.50	28.90	32.10
	**14**	9.48	9.00	10.02	155.20	149.41	168.59	2.02	2.04	2.03	36.30	34.60	36.30
**Benzene**	**0**	0.00	0.00	0.00	0.00	0.00	0.00	3.07	3.07	3.07	11.00	13.70	11.70
	**7**	1.07	1.29	1.71	7.93	4.13	4.34	2.82	2.81	2.81	28.00	31.00	27.00
	**14**	1.29	1.98	1.82	10.02	5.46	6.48	2.72	2.72	2.71	31.00	32.00	30.00
**Methanol**	**0**	0.00	0.00	0.00	0.00	0.00	0.00	1.25	1.25	1.25	11.00	13.70	11.70
	**7**	1.59	1.39	1.07	7.71	4.88	4.61	0.52	0.52	0.51	32.00	31.00	37.00
	**14**	1.18	1.71	1.88	6.59	5.46	4.66	0.16	0.17	0.16	37.00	38.00	43.00

Table S2. Identification of MCT, DH, and SAM genes in the genome and the primers designed for qPCR analysis. The search for protein sequences of hits with query coverage≥40%, sequence identity≥20% and e-value≤1×10^{-5} threshold were extracted to obtain a set of homologs (79). Reference gene were *Act1* and *tef1*. Definition of abbreviations, *mct*=methyl chloride transferase, *dh*=dehalogenase, *ms*=methionine synthetase. The Numbers after the abbreviations represent different genes.

	GenBank accession numbers	Gene name	Reference gene ID	Identity*	Coverage*	*E*-value	Abbr	Primer - F (5' to 3')	Primer - R (5'to 3')
S. commune* *20R-07-F01	OM864274	Methyl chloride transferase	ESK88094.1	47%	94%	4.23E-55	*mct1*	ATTCCTGGGATATTGCATGG	GTCGAATATGCGAGTTAGCTC
	OM864275	Methyl chloride transferase	ESK88094.1	42%	95%	6.41E-47	*mct2*	GCTGCGAGAGATCGTCG	GGTATGCGACATCGTTTCC
	OM864268	Methionine synthetase	P42319	79%	58%	5.00E-151	*ms*	CAAGACGGGCATGATCATGG	AACATGATACCCTGGTCACC
	OM864272	Dehalogenase	RJE21457.1	50%	96%	7.00E-87	*dh1*	CGGTACGTAGTGACATCC	TGGCTTCTTCTTGGGTCC
	OM864269	Dehalogenase	TFK65948.1	58%	97%	6.00E-92	*dh2*	TGGTACGGCACGATCAAG	TCCCTAACGAAGGAGTGC
	OM864270	Dehalogenase	ATC24116.1	72%	99%	4.00E-180	*dh3*	TCACTCCCCAGCCCAACC	GCTTGTCCGTCTCGCCG
	OM864271	Dehalogenase	WP_019862921.1	51%	89%	6.00E-102	*dh4*	GCTTCATCCCTGCTGCAC	CTGGTCCGTTGGATGATAGC
	OM864273	Dehalogenase	WP_019862921.1	56%	81%	3.00E-120	*dh5*	TCAAGTATACCTACCACTACGC	GCTTGTCCGTGCCTCC
		Reference genes					*act1*	TGGTATCCTCACGTTGAAGTA	GTGTGGTGCCAGATCTT

							tef1	AGCTTGGCAAGGGTTCCTTCA	AACTTCCAGAGGGCGATATCA
Agaricus bisporus	XP_006463659.1	Methyl chloride transferase	OM864274	48%	946%	5.00E-57	*mct1*	GATCTTGTTTACGATTATACGTTTTTC	GTTATGAGGTATCCTCCAGGTT
	XP_006460446.1	Dehalogenase	OM864270	22%	79%	6.24E-24	*dh3*	GCGTACATCCGTTGCCAG	CGGAGGAAGGGTCTTGTTG
	GCA_000300575.1	Dehalogenase	OM864271	49%	95%	7.00E-87	*dh4*	GGATCGCAAAGCAATTCTAGC	GTAGTTGCCGAGCCTGAATTTC
	XP_006454629.1	Dehalogenase	OM864273	36%	99%	6.54E-78	*dh5*	CTGGCGTTCATCTGTCGTCA	AAACGGAAGAAGGGTCTTGCAA
	SOP81487.1	Methionine synthetase	OM864268	89%	99%	6.23E-21	*ms*	CAAGACGGGCATGATCATGG	AACATGATACCCTGGTCACC
		Reference genes					*act1*	TGGTATCCTCACGTTGAAGTA	GTGTGGTGCCAGATCTT
Hypsizygus marmoreus	RDB16040.1	Methyl chloride transferase	OM864274	48%	96%	2.00E-59	*mct1*	CGTCAATGTCAAGTTCAAACTTG	ACCAGGCCTGATCAAGGAC
	GCA_000300575.1	Dehalogenase	OM864270	52%	93%	3.00E-109	*dh3*	CCACAGTCAAGCTAGAGGATG	CAGGTCGATCATCTGCCAAAC
	GCA_000300575.1	Dehalogenase	OM864271	23%	47%	6.41E-47	*dh4*	ATGGAGGCTGTCGAAGCTC	TGTACCCATTCCTCCATTCTTG
	RDB19886.1	Dehalogenase	OM864273	32%	86%	9.15E-33	*dh5*	GTATATGGGACACTTGTGGACT	TACGGCATCTCTGGGTATTGC
	RDB28953.1	Methionine synthetase	OM864268	36%	96%	1.20E-87	*ms*	CAAGACGGGCATGATCATGG	AACATGATACCCTGGTCACC
		Reference genes					*act1*	TGGTATCCTCACGTTGAAGTA	GTGTGGTGCCAGATCTT
Pleurotus ostreatus	KDQ29133.1	Methyl chloride transferase	OM864274	45%	97%	6.00E-57	*mct1*	ATTCCTGGGTTATTGCATCG	GTCGATTATGCGAGTTTGGTC
	KDQ22839.1	Dehalogenase	OM864270	26%	40%	2.26E-1	*dh3*	CATTCAAGCATTCATCTTCGATGTGTT	CAATCTCATTTGTTGCGACCCA

					0			
KDQ30942.1	Dehalogenase	OM864271	43%	97%	1.03E-94	*dh4*	GCGTGCCGTTTCGCTTCTG	CTGACTCCCAGTGACTGC
KDQ28170.1	Dehalogenase	OM864273	44%	96%	5.47E-99	*dh5*	CGAGGGGCTCATTCCAATTATC	CATTTGACGGTCCAGTTCCTTG
KDQ30862.1	Methionine synthetase	OM864268	37%	99%	3.74E-81	*ms*	CAAGACGGGCATGATCATGG	AACATGATACCCTGGTCACC
	Reference genes					*act1*	TGGTATCCTCACGTTGAAGTA	GTGTGGTGCCAGATCTT

Table S3. The relative quantity (RQ) of gene expression of strain 20R-7-F01 in methanogenic substrates (glucose, lignin, phenol, and anisic acid) compared to non-methanogenic substrates methanol and benzene. RQ values were calculated by the $2^{-\Delta\Delta CT}$ method (75). Compared with non-methanogenic substrates, the expression of the target gene was up-regulated only when RQ>2 and *p*-value<0.05 (64). *mct*=methyl chloride transferase, *dh*=dehalogenase, *ms*=methionine synthetase. The Numbers after the abbreviations represent different genes.

**Non-methanogenic substrate**	**Methanogenic substrate**	***mct1***		***mct2***		***ms***		***dh1***		***dh2***		***dh3***		***dh4***		***dh5***	
		**RQ**	***p*-value**	**RQ**	***p*-value**	**RQ**	***p*-value**	**RQ**	***p*-value**	**RQ**	***p*-value**	**RQ**	***p*-value**	**RQ**	***p*-value**	**RQ**	***p*-value**
Benzene	Phenol	2.23	0.01	1.29	0.26	2.14	0.01	2.25	0.92	0.95	0.8	4.18	1.61E-05	3.64	5.52E-05	3.05	3.88E-03
	p-Anisic acid	2.32	1.56E-03	1.22	0.55	2.28	0.02	0.04	0.25	1.14	0.46	6.32	5.03E-04	3.26	0.01	4.69	3.88E-03
	Lignin	2.84	3.41E-04	0.96	0.34	2.97	0.01	0.34	0.36	1.14	0.39	8.83	0.01	2.67	0.01	4.21	3.59E-04
	Glucose	2.46	2.50E-03	1.69	0.01	2.66	3.64E-03	1.47	0.44	1.1	0.49	6.77	1.87E-03	3.96	3.65E-03	3.93	0.01
Methanol	Phenol	2.08	0.02	0.88	0.31	2.03	1.44E-03	4.24	0.1	1.07	0.92	2.31	2.04E-03	3.78	4.23E-05	2.23	1.90E-03
	p-Anisic acid	2.16	3.08E-03	1.1	0.17	2.17	0.01	0.07	0.06	1.28	0.41	3.5	0.01	3.38	4.52E-03	3.44	0.01
	Lignin	2.65	1.18E-03	0.66	0.01	2.82	0.01	0.64	0.32	1.28	0.4	4.88	0.04	2.77	0.01	3.08	1.54E-03
	Glucose	2.29	0.01	1.15	0.14	2.53	3.64E-03	2.78	0.07	1.23	0.47	3.74	0.01	4.11	3.36E-03	2.88	0.01

Table S4. The relative quantity (RQ) of *dhs* expression in strain 20R-7-F01 in 200 nmol and 5,400 nmol exogenous CH_3Cl compared to that in CH_3Cl free control. RQ values were calculated by the $2^{-\Delta\Delta CT}$ method (75). Compared with none CH_3Cl, the expression of the target gene (in CH_3Cl) was up-regulated only when RQ>2 and p-value<0.05 (64). *dh*=dehalogenase. The Numbers after the abbreviations represent different genes.

**Non exogenous CH_3Cl**	**Exogenous CH_3Cl (nmol)**	***dh1***		***dh2***		***dh3***		***dh4***		***dh5***	
		**RQ**	***p*-value**	**RQ**	***p*-value**	**RQ**	***p*-value**	**RQ**	***p*-value**	**RQ**	***p*-value**
0	200	0.14	0.24	0.96	0.04	3.63	1.00E-03	1.20	0.45	0.02	0.39
	5,400	0.59	0.33	1.34	0.23	11.23	3.00E-05	1.78	0.37	0.34	0.46

Table S5. Production of CH_4 and CH_3Cl by three wood rot fungi in methanogenic substrate *p*-anisic acid and non-methanogenic substrate benzene, and the relative quantity (RQ) of gene expression of methane-related genes in *p*-anisic acid compared to benzene. RQ values were calculated by the $2^{-\Delta\Delta CT}$ method (75). Compared with non-methanogenic substrates, the expression of the target gene was up-regulated only when RQ>2 and *p*-value<0.05 (64). *mct*=methyl chloride transferase, *dh*=dehalogenase, *ms*=methionine synthetase. The Numbers after the abbreviations represent different genes.

**Species**	**Substrate**	**CH_4 (nmol)**	**CH_3Cl (nmol)**	***mct1***		***ms***		***dh3***		***dh4***		***dh5***	
				**RQ**	***p*-value**	**RQ**	***p*-value**	**RQ**	***p*-value**	**RQ**	***p*-value**	**RQ**	***p*-value**
Agaricus bisporus	Benzene	0.40 ± 0.24	16.79 ± 5.60										
	p-Anisic acid	1.53 ± 0.51	107.54 ± 16.04	2.45	5.54E-05	2.36	4.27E-03	2.95	4.53E-02	2.06	6.88E-07	114.46	5.79E-03
Hypsizygus marmoreus	Benzene	0.11 ± 0.11	10.58 ± 4.79										
	p-Anisic acid	1.88 ± 0.32	131.19 ± 8.14	14.11	1.80E-05	13.55	1.06E-04	6.26	1.54E-05	17.03	1.43E-04	8.8	1.11E-04
Pleurotus ostreatus	Benzene	0.29 ± 0.29	13.58 ± 5.33										
	p-Anisic acid	1.61 ± 0.16	123.64 ± 13.61	2.79	0.02	2.22	3.81E-04	17.13	7.25E-06	20.78	0.98	0.88	1.61E-04
**Control**	**CK**	0.38 ± 0.16	8.30 ± 1.71										

SUPPLEMENT TEXT

1. Procedure for anaerobic cultivation of fungi

The method of Miller and Wolin (1974) was adopted to conduct anaerobic cultivation of fungi (60), with minor modifications. Liquid media prepared by this method remain anoxic, as indicated by resazurin reduction, at room temperature for up to 6 months (60). The cultivation process is as follows:

Step 1. Anaerobic test strips (Thermo; BR0055B; pO_2 detection limit≥0.2%) (61) were fixed to inner walls in the uppermost portion of cultivation bottles (140 mL) with heat-resistant adhesive. Aliquots of media (20 ml per bottle) were added. Butyl rubber stoppers (Jinling, 28#, septa thickness: 8 mm) were placed into a beaker. Bottles containing media and the beaker were sealed with a breathable sealing film (BKMAN, China) and sterilized by autoclaving at 121℃ for 20 minutes. Afterward, the anaerobic strip appeared pink, indicating oxic conditions (62). All media bottles were stored at 85℃ and taken out one by one to carry out the following operations.

Step 2. For O_2 removal, the hot media were purged with O_2-free N_2 for 15 min while the medium was cooling. All flushing was done using a gas manifold fitted with disposable hypodermic needles that were equipped with a syringe filter (BKMAN, 0.22 μm, China) above the needle to prevent contamination with microorganisms from the N_2 stream. The outlet of the needle was inserted at the bottom of the media to ensure effective purging of O_2 by bubbling with nitrogen. After 12~15 min of purging, the indicator color had changed from pink to white indicating anoxic conditions.

Step 3. Fungi and filtered trace elements were subsequently added under continued purging with O_2-free N_2 (80). Subsequently, media were purged with N_2 for at least 5

additional minutes.

Step 4. Bottles were sealed with autoclaved butyl stoppers. To avoid O_2 intrusion, stoppers were carefully inserted into the mouths of bottles and needles used for N_2-flushing slowly withdrawn.

Step 5. All bottles were crimped with aluminum caps to stoppers, and additionally sealed with wax to prevent outside stopper contact with air (81).

Step 6. Gas syringes (Hamilton, 1 mL, USA) equipped with needles (Hamilton, OD 0.63 mm, USA) and valves were used to draw out gas samples. Needles were flushed 3 times with N_2 before and after each use. Each bottle was only pierced and sampled once.

2. No detectable O_2 content in headspace of culture bottles

The results of gas chromatographic analysis showed that pO_2 content in the bottle headspace (120 mL) was less than 0.2% (4.5 μM medium concentration) when the anaerobic test strip turned white completely (about 10 min, Fig. S6A). Conservatively assuming that O_2 concentrations were right at the threshold of the anaerobic test strip (In fact, the bottle is flushed with pure N_2 for at least another 10 minutes after the indicator turns white), the total amount of O_2 per bottle was 11.22 μmoL. *[Note: the GC method* (82) *involved an HP-PLOT 5A column (30 m×0.32 mm×25 μm) (Agilent, USA) with a TCD detector and helium as a carrier gas (5 mL L^{-1}). Operating temperatures were 60 ℃ for the injection port, 40 ℃ for the column and 180 ℃ for the thermal conductivity detector.]*

Moreover, strain 20R-7-F01 consumed 40~930 μmoL of organic carbon from *p*-anisic acid, glucose, lignin, lignite over 7 days (Table S1), and 50~1330 μmoL carbon

over 14 days (Table S1). Under aerobic respiration, which *S. commune* is capable of, oxygen (O_2) and organic carbon would be consumed at a 1:1 stoichiometry (83). Thus, the O_2 demand for aerobic growth greatly exceeded the maximum amount of O_2 (11.22 µmoL) that was present under the most conservative scenario, and it is clear that fungal growth and metabolism were predominantly fueled by anaerobic processes.

3. The addition of reductants in the medium had no effect on methane production by the fungus

To rule out trace O_2 requirements for fungal growth, control media containing significant concentrations of effective O_2-scavenging reductants (1 or 2 mM iron chloride ($FeCl_2$) or 0.6 mM ascorbic acid) were prepared. Addition of chemical reductants had no significant impact (p=0.36~0.54, abscissa in blue font) on methane production by the fungus (Fig. S6B). While methane production also occurred under an oxic headspace (20% O_2 and 80% N_2), suggesting that *S. commune* is also capable of aerobic methane production, the amount of methane was significantly lower (28.5%; p=0.003, none/Aerobic and none/Anaerobic) in the presence of O_2 (Fig. S6B).
